# Crystal structures of two novel iron isocyanides from the reaction of 2,6-di­methyl­phenyl isocyanide, CNXyl, with bis­(anthracene)ferrate(−1)

**DOI:** 10.1107/S205698902101313X

**Published:** 2022-01-01

**Authors:** William W. Brennessel, John E. Ellis

**Affiliations:** a Department of Chemistry, 120 Trustee Road, University of Rochester, Rochester, NY 14627, USA; b Department of Chemistry, 207 Pleasant Street SE, University of Minnesota, Minneapolis, MN 55455, USA

**Keywords:** crystal structure, 2,6-di­methyl­phenyl isocyanide, xylylisocyanide, CNX­yl, iron, infrared spectroscopy

## Abstract

The reaction of the [K(18-crown-6)(thf)_2_]^1+^ (thf is tetra­hydro­furan) salt of bis­(anthracene)ferrate(−1), or [Fe(C_14_H_10_)_2_]^−^, with 2,6-di­methyl­phenyl isocyan­ide (CNX­yl) in thf resulted in the formation of two new iron isocyanide complexes, namely, [(1,2,3,4-η)-anthracene]tris­(2,6-di­methyl­phenyl isocyanide)iron, [Fe(C_14_H_10_)(C_9_H_9_N)_3_] or [Fe(1,2,3,4-η-C_14_H_10_)(CNX­yl)_3_], and {5,6-bis­(2,6-di­methyl­anilino)-3-(2,6-di­methyl­phen­yl)-1,2,7-tris­[(2,6-di­methyl­phen­yl)imino]-3-azoniahept-3-ene-1,4,7-triido}tris­(2,6-di­methyl­phenyl isocyanide)iron tetra­hydro­furan disolvate, [Fe(C_54_H_56_N_6_)(C_9_H_9_N)_3_]·2C_4_H_8_O or [Fe(C_54_H_56_N_6_)(CNX­yl)_3_]·2C_4_H_8_O, which were characterized by single-crystal X-ray diffraction. The former is likely an inter­mediate along the path to the known homoleptic [Fe(CNX­yl)_5_], while the latter contains a tridentate ligand that is formed from the ‘coupling’ of six CNXyl ligands. A third crystal structure from this reaction, (7-methyl­indol-1-ido-κ*N*)(1,4,7,10,13,16-hexa­oxa­cyclo­octa­decane-κ^6^ *O*)potassium, [K(C_9_H_8_N)(C_12_H_24_O_6_)] or [K(C_9_H_8_N)(18-crown-6)], contains a 7-methyl­indol-1-ide anion, in which one CNXyl ligand has shed a proton during its reductive cyclization.

## Abstract 2

Three crystal structures from the reaction of 2,6-di­methyl­phenyl isocyanide with bis­(anthracene)ferrate(−1) are presented.

## Chemical context

The low-valent bis­(anthracene)cobaltate(−1) has been shown to be an excellent source of spin-paired atomic Co(−1) anions in substitution reactions in which both anthracene (C_14_H_10_) ligands are readily displaced by a wide variety of acceptor ligands (Brennessel *et al.*, 2002 ▸; Brennessel & Ellis, 2012 ▸). The reaction with four equivalents of CNXyl, Xyl is 2,6-di­methyl­phenyl, resulted in an excellent yield of the homoleptic isocyanidecobaltate(−1), [Co(CNX­yl)_4_]^1−^, first obtained by an alternate synthesis (Warnock & Cooper, 1989 ▸). Attempts to prepare the analogous 18-electron iron complex, bis­(anthra­cene)ferrate(−2), afforded only the related 17-electron, paramagnetic bis­(anthracene)ferrate(−1) (Brennessel *et al.*, 2007 ▸). The latter species was shown to react with carbon monoxide to afford excellent yields of the Fe(−1) complex, [Fe_2_(CO)_8_]^2−^. On this basis, the corresponding reaction with CNXyl in tetra­hydro­furan, thf, was examined to determine whether the unknown [Fe_2_(CNX­yl)_8_]^2−^ could be accessed. Bis(anthracene)ferrate(−1) was also reacted with excess CNXyl in the presence of one equivalent of a reducing agent to see whether the previously reported monometallic [Fe(CNX­yl)_4_]^2−^ (Brennessel & Ellis, 2007 ▸) could be prepared by this facile route. However, in both cases, infrared (IR) spectroscopy indicated predominant formation of the long-known, but only recently structurally authenticated Fe^0^ complex, [Fe(CNX­yl)_5_] (Bassett *et al.*, 1979 ▸, Brennessel *et al.*, 2019 ▸). 
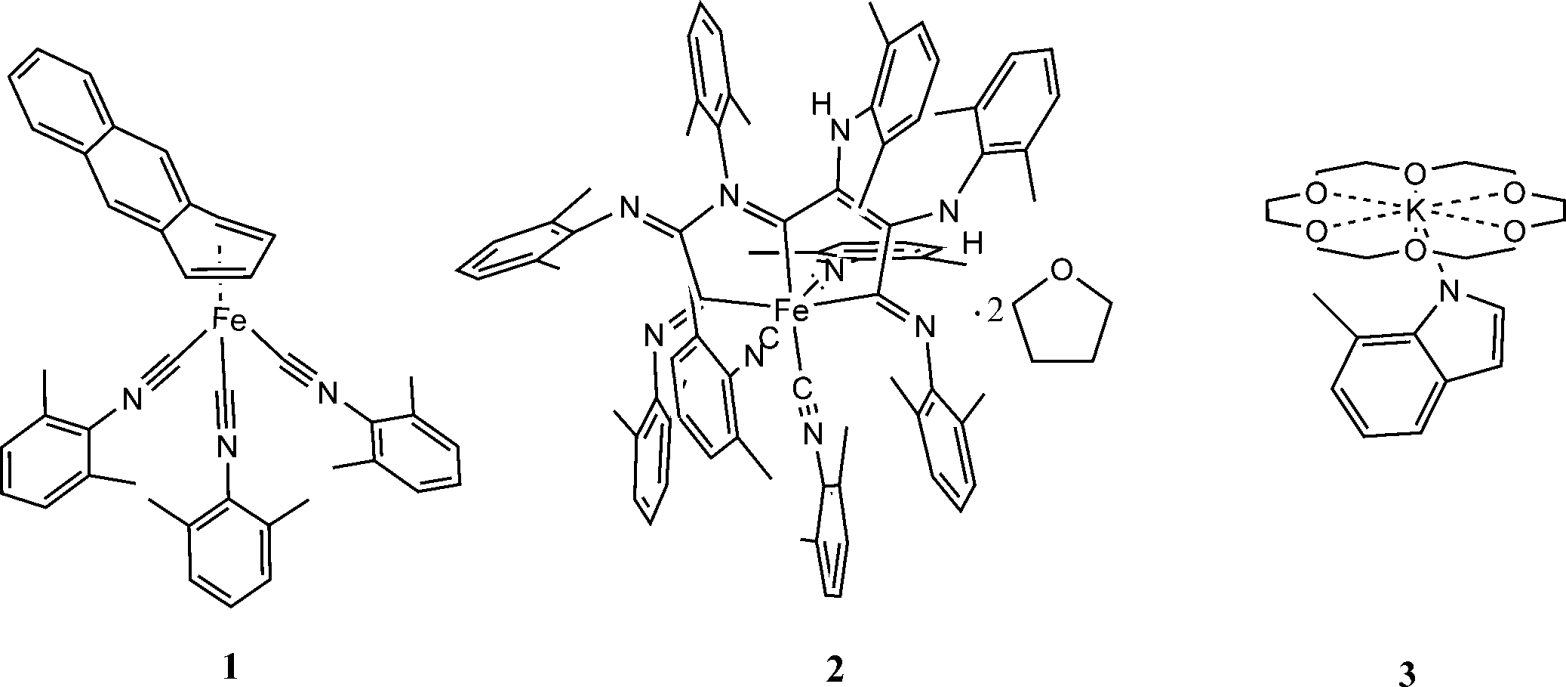


**Figure 1:**
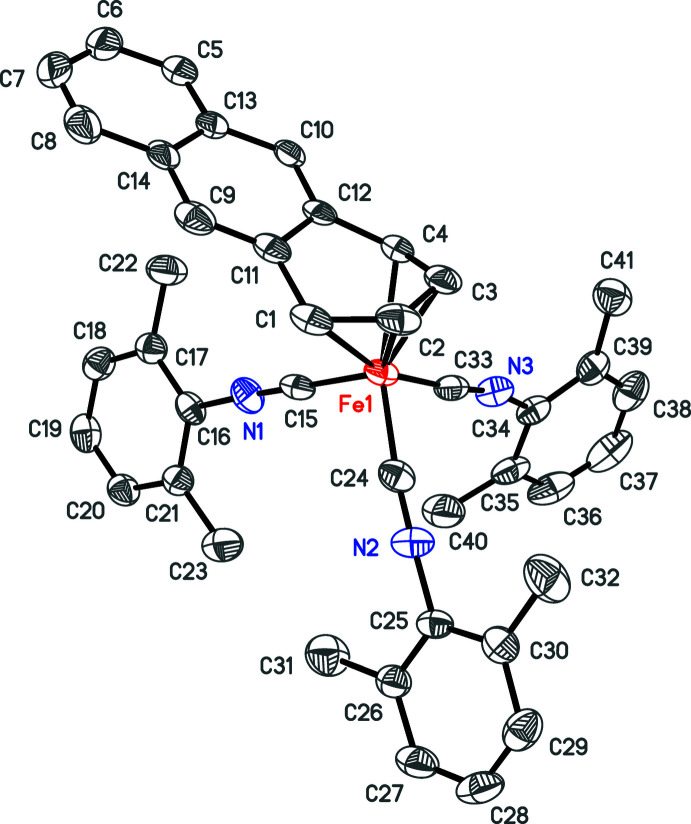
Anisotropic displacement ellipsoid plot of **1** drawn at the 50% probability level with H atoms omitted.

Because a complex containing formally Fe^-I^ resulted in an oxidation to Fe^0^, it was of inter­est to determine what other species were produced by the reaction of bis­(anthracene)ferrate(−1) with excess CNXyl in THF. First an aliquot was taken from the reaction mixture early on and placed in a 243 K freezer until orange blocks were observed. A single crystal X-ray diffraction experiment revealed these to be [Fe(C_14_H_10_)(CNX­yl)_3_] **1** (Fig. 1 ▸). It is thought that this complex is likely a crystallization-trapped inter­mediate, since [Fe(CNX­yl)_5_] is ultimately produced. Compound **1** is of inter­est as the first mixed anthracene–isocyanide derivative of the unknown bis­(anthracene)iron(0). However, the related carbonyl, [Fe(C_14_H_10_)(CO)_3_], has been known for more than 50 years (Manuel, 1964 ▸).

**Figure 2:**
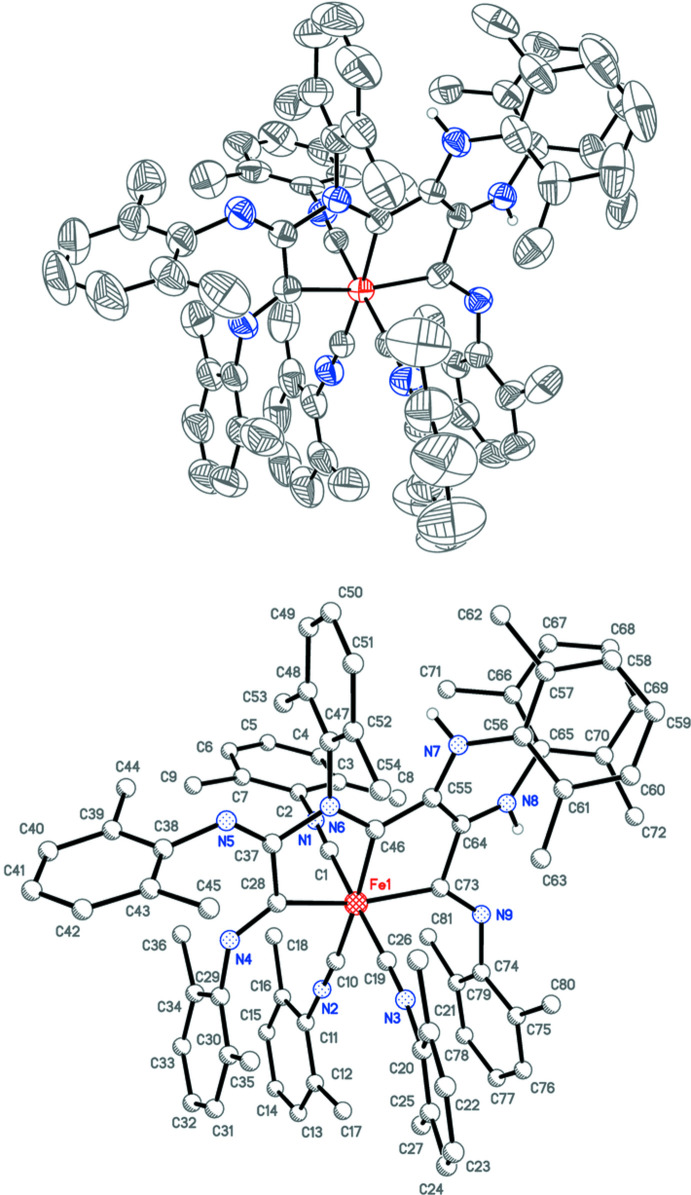
Plots of **2** with C—H hydrogen atoms and solvent mol­ecules omitted and with only the major components of disorder shown. Top: anisotropic displacement ellipsoid plot drawn at the 50% probability level. Bottom: ball-and-stick plot in the same orientation featuring the numbering scheme.

**Figure 3:**
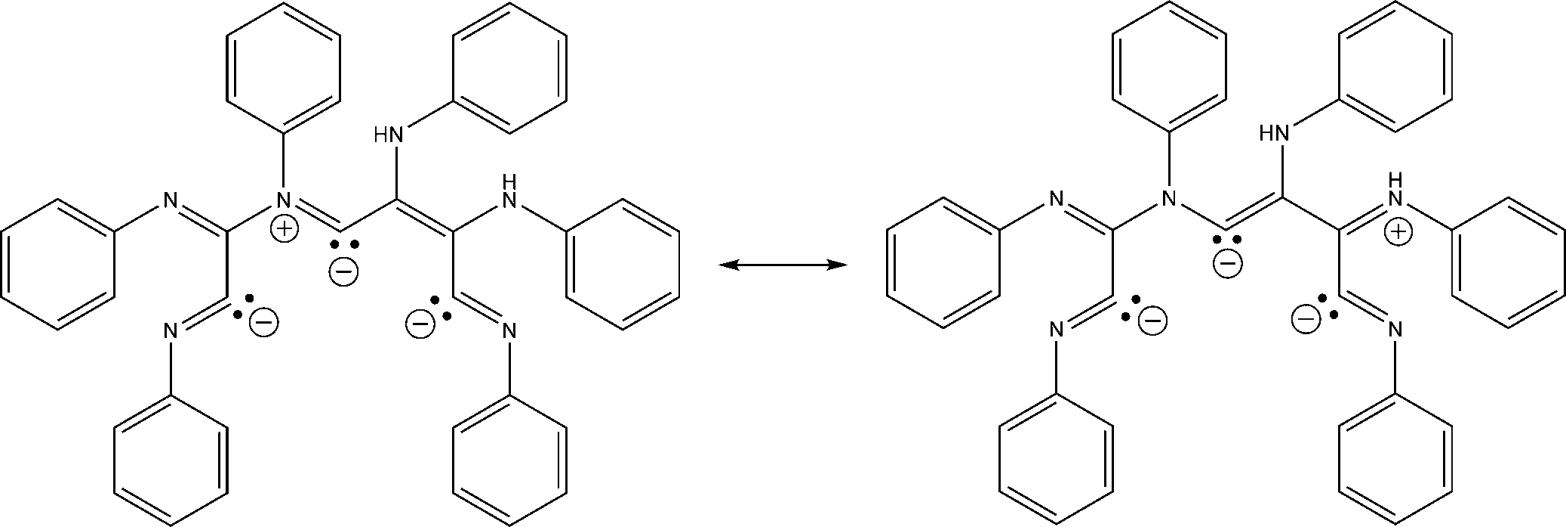
Two proposed resonance forms of the tridentate dianion of **2** based on the bond lengths.

After the reaction mixture had warmed to room temperature and stirred for a few hours, the solvent was removed and *n*-heptane was added. The mixture was then filtered and a new species crystallized in the filtrate. A crystal structure revealed the material to be a thf disolvate of [Fe(C_54_H_56_N_9_)(CNX­yl)_3_] **2** (Fig. 2 ▸). In this case, six isocyanides had reductively ‘coupled’ to form a previously unknown tridentate ligand that had been protonated twice at two of the nitro­gen atoms (Fig. 3 ▸). An IR spectrum obtained from the few crystals that could be harvested showed νCN stretches of 2110*w* and 2055*vs* cm^−1^, consistent with an Fe^+2^ oxidation state, which would make the ligand formally dianionic. The source of the protons in aprotic media was still a mystery at this point.

**Figure 4:**
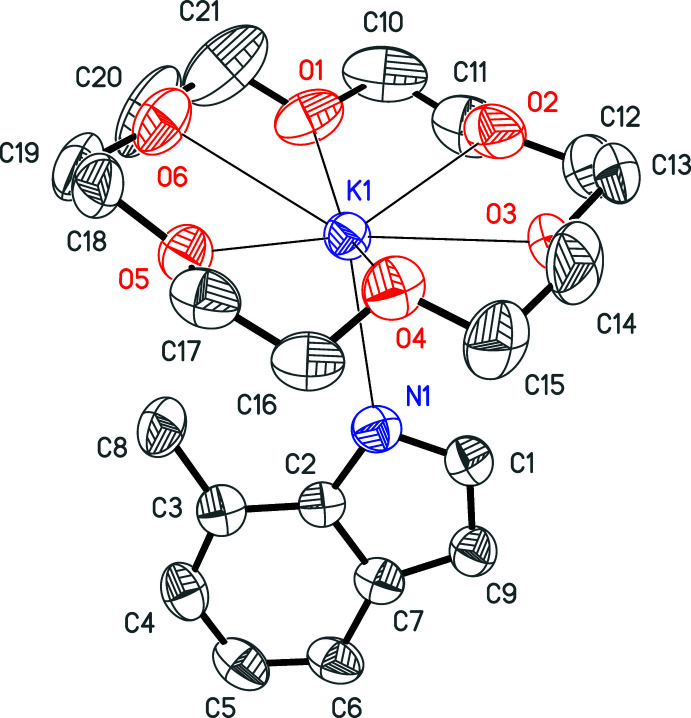
Anisotropic displacement ellipsoid plot of **3** drawn at the 50% probability level with H atoms and the minor component of disorder omitted.

Coupling of isocyanide ligands has precedent (Yamamoto & Yamazaki, 1972 ▸, Lam *et al.*, 1977 ▸, Giandomenico *et al.*, 1982 ▸, Warner & Lippard, 1986 ▸), although this exact ‘coupling’ of six isocyanide ligands appears to be new. The protonation of nitro­gen atoms has also been observed in such circumstances. For instance, reduction of [Mo(CN*R*)_6_ *X*]^+^ (many variations on *R* and *X*) by Zn in the presence of water generated a bis(alkyl­amino)­acetyl­ene ligand with protonated nitro­gen atoms (Lam *et al.*, 1977 ▸; Giandomenico *et al.*, 1982 ▸; Warner & Lippard, 1986 ▸). The source of protons in the production of **2**, however, was not discovered until single crystals grown from the heptane-insoluble component were evaluated. The structure was formulated by X-ray diffraction as [K(18-crown-6)(C_9_H_8_N)] **3** (Fig. 4 ▸), a cyclized, reduced form of CNXyl, from which one hydrogen atom was lost. It must be emphasized that examples of trimerization (Yamamoto *et al.*, 1982 ▸; Blake *et al.*, 1997 ▸; Bashall *et al.*, 2000 ▸; Chen *et al.*, 2019 ▸), tetra­merization (Shen *et al.*, 2014 ▸; Altenburger *et al.*, 2016 ▸; Kucera *et al.*, 2019 ▸), penta­merization (Tanase *et al.*, 1992 ▸, 1996 ▸), hexa­merization (Shen *et al.*, 2014 ▸), and polymerization (Yamamoto & Yamazaki, 1972 ▸) of isocyanides are well-precedented, but **2** appears to be only the second example in which hexa­merization of an organic isocyanide has been established.

Given the speciation observed by the crystal structures and IR spectroscopy, a balanced equation can be written [Equation (1)]. The hydrogen atom lost during the reduction and cyclization that forms **3** is now found in the two protonations in the one-half equivalent of **2**.

Inter­estingly, in support of this equation, when less than eight equivalents of CNXyl were employed (*e.g*., four), intra­ctable tars resulted. It should be noted, however, that this equation is only speculative and requires further investigation for confirmation.

**Equation (1)**

[K(18-crown-6)(thf)_2_][Fe(C_14_H_10_)_2_] + 8 CNXyl → 0.5 [Fe(CNX­yl)_5_] + 0.5 [Fe(C_54_H_56_N_6_)(CNX­yl)_3_] + [K(18-crown-6)(C_9_H_8_N)]

## Structural commentary

The geometry at the formally zerovalent iron center of **1** is nearly identical to those of related mol­ecules with one 1,2,3,4-η-naphthalene *o*-anthracene ligand and three excellent acceptor ligands in a tripodal arrangement. The average of the three (XylN)C—Fe—C(NX­yl) angles of **1**, 95.3°, matches well with that of the average C—Fe—C angle from three carbonyl ligands of the [Fe(1,2,3,4-η-naphthalene)(CO)_3_] portion of a trinuclear mol­ecule, 97.5° (Imhof, 1999 ▸), and those of the average P—Fe—P angles from [Fe(1,2,3,4-η-naphthalene)(P(OMe)_3_)_3_], 97.7° (Schäufele *et al.*, 1989 ▸), and [Fe(1,2,3,4-η-anthracene)(P(OMe)_3_)_3_], 97.9° (Brennessel *et al.*, 2007 ▸). The ‘fold angle’ between the iron-coordinating η^4^-diene unit and the exo-benzene or -naphthalene portions are 30.7, 30.2, 40.6, and 40.8°, respectively, for the same four structures. The latter two angles are significantly larger than those in mol­ecules containing three CNXyl or CO ligands, and since the Fe—C(η^4^-diene) bond lengths (Table 1 ▸) in all four structures are comparable, it would be inter­esting to know if this is an electronic effect due to the different nature of CO/CNXyl versus phosphite and/or due to the bulk of the tri­methyl­phosphite ligands.

The ligand set of **2** is built from nine CNXyl ligands, of which six, with the addition of two protonations at nitro­gen atoms, have joined together into one tridentate dianionic ligand. Because this ligand is essentially planar at the core of two fused metalla­cyclo­penta­nes (Fig. 2 ▸), it binds the iron center meridionally. The three remaining CNXyl ligands are also meridional, resulting in a distorted octa­hedral geometry. The bond lengths in the fused ring core (Table 2 ▸) suggest resonance stabilization (Fig. 3 ▸). To our knowledge, only one other ‘coupling’ of six isocyanide ligands has been structurally verified. In this case, six cyclo­hexyl isocyanide ligands have ‘coupled’ into a dianionic ligand (without any protonations) that bridges two chromium centers (Shen *et al.*, 2014 ▸).

In **3**, one CNXyl mol­ecule has reductively cyclized into a 7-methyl­indol-1-ide anion (Fig. 4 ▸). The potassium cation is inter­acting normally with an 18-crown-6 macrocycle, and additionally with the nitro­gen atom of the anion (Table 3 ▸).

## Supra­molecular features

**Figure 5:**
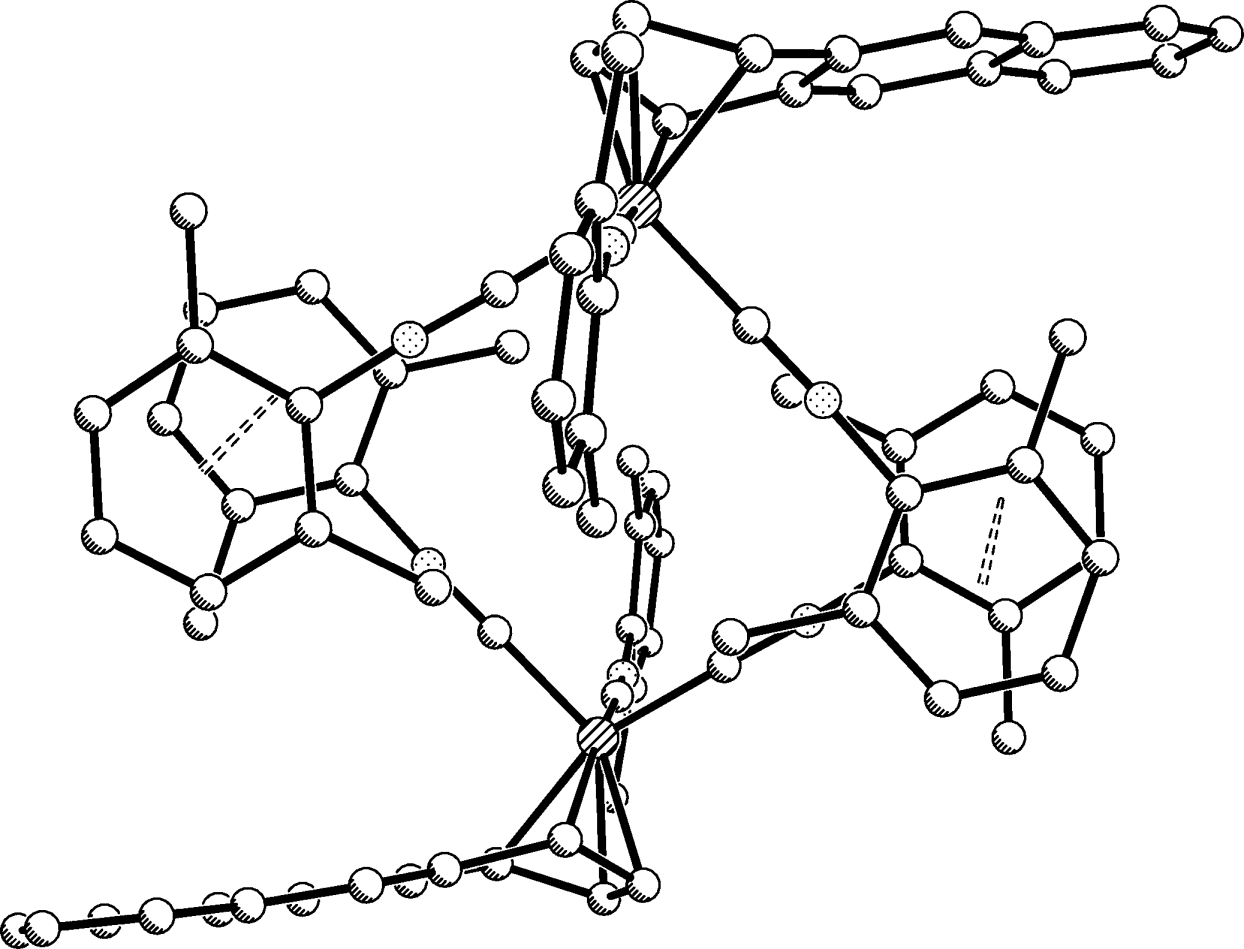
Depiction of the offset parallel π–π inter­actions between two mol­ecules of **1** whose centroid–centroid (dashed lines) distances are 3.59 Å. The second mol­ecule is generated by inversion operator 1 − *x*, 1 − *y*, −*z*.

In addition to several inter­molcular edge-to-face (C—H⋯π) inter­actions, pairs of mol­ecules in **1** are linked by offset parallel (slippage, 0.85 Å) π–π inter­actions (Fig. 5 ▸), whose centroid–centroid distances are 3.588 (2) Å. In **2** there is one instance of an intra­molecular offset parallel (slippage, 1.24 Å) π–π inter­action between phenyl rings C56–C61 and C65–C70 [Fig. 2 ▸, centroid–centroid distance, 3.614 (9) Å]. The acceptor for the N7—H7 donor is the π-system of phenyl ring C47–C52 and that for the N8—H8 donor is intra­molecular acceptor N9 (Table 4 ▸). No obvious inter­molecular inter­actions are observed in **2**, which may also explain the reason for the significant disorder in the thf mol­ecules (*i.e*., there are no C—H⋯O inter­actions from the iron complex to anchor them). The inter­molecular inter­actions in **3** are limited to C—H⋯π inter­actions between methyl­ene hydrogen atoms and the indenyl π-system.

## Synthesis and crystallization

All manipulations were carried out under argon using standard Schlenk techniques to maintain strictly anaerobic conditions. Solvents were dried using standard techniques, as described previously (Brennessel & Ellis, 2012 ▸). [K(18-crown-6)(THF)_2_][Fe(C_14_H_10_)_2_] and CNXyl were prepared according to previously reported procedures (Brennessel *et al.*, 2007 ▸ and Brennessel *et al.*, 2019 ▸, respectively).

To a deep-orange solution of [K(18-crown-6)(thf)_2_][Fe(C_14_H_10_)_2_] (1.000 g, 1.163 mmol) in thf (100 mL, 195 K) was added CNXyl (1.373 g, 10.47 mmol) in thf (40 mL, 195 K). The reaction mixture was warmed slowly to room temperature. A solution IR spectrum showed no anionic species, but a broad peak with shoulders that matched the well-known [Fe(CNX­yl)_5_] (Bassett *et al.*, 1979 ▸), as well as a sharp peak for free CNXyl. An aliquot taken early in the reaction was placed in a freezer (243 K), from which orange crystals of **1** were structurally determined. The solvent was removed from the main reaction mixture and heptane was added with vigorous stirring. Crystals grown from the filtrate (*i.e*., heptane-soluble component) were identified as **2** by X-ray diffraction. IR spectroscopy on the crystals (Nujol mull) gave νCN = 2110*w* and 2055*vs* cm^−1^. The filter cake (*i.e*., heptane-insoluble component) was redissolved in THF and layered with pentane, which resulted in crystals of **3** as determined by a single crystal X-ray experiment. No characterization beyond what is presented above was performed.

## Refinement

Crystal data, data collection and structure refinement details are summarized in Table 5 ▸. Intensity data for **2** were collected at 293 (3) K. A preliminary collection at 173 (2) K resulted in a primitive monoclinic cell which was modulated such that the *b*-axis was doubled and the *a*-axis could be determined as multiples of approximately 13 Å, the best being 52 Å.

In **2**, two CNXyl groups were modeled as disordered over two positions each: N1/C1–C9, 0.52 (2):0.48 (2) and N8/C64–C72, 0.57 (2):0.43 (2). Additionally, the two THF solvent mol­ecules were modeled as disordered over two positions each: O1/C82–C85, 0.55 (2):0.45 (2) and O2/C86–C89, 0.69 (1):0.31 (1).

In **3**, the anion is modeled as disordered with a planar flip of itself [0.905 (3):0.095 (3)]. The 18-crown-6 macrocycle is also disordered in a similarly lopsided component ratio; the eight largest residual peaks are the two peaks near the K atom and those for six O atoms of the minor component of disorder. However, the data-to-parameter ratio drops below eight if this disorder is modeled. Thus only the anion disorder was modeled.

To model the various disordered species, analogous bond lengths and angles were restrained to be similar and anisotropic displacement parameters for proximal atoms were restrained to be similar. For the THF solvent mol­ecules in **2**, bond lengths were restrained toward ideal values and anisotropic displacement parameters were additionally restrained toward the expected motion relative to bond direction.

The H atoms on the metal-coordinating carbon atoms (C1–C4) of **1** were refined freely to confirm their nature and better describe their true positions. In **2**, H7 was also refined freely. All other H atoms were placed geometrically and treated as riding atoms. For **1** and **3** (173 K), methyl­ene, C—H = 0.99 Å, aromatic/*sp* ^2^, C—H = 0.95 Å, with *U*_iso_(H) = 1.2*U*_eq_(C), and methyl, C—H = 0.98 Å, with *U*_iso_(H) = 1.5*U*_eq_(C). For **2** (293 K), methyl­ene, C—H = 0.97 Å, aromatic/*sp* ^2^, C—H = 0.93 Å, N—H = 0.86 Å, with *U*_iso_(H) = 1.2*U*_eq_(C), and methyl, C—H = 0.96 Å, with *U*_iso_(H) = 1.5*U*_eq_(C).

For **1** the maximum residual peak of 0.36 e^−^ Å^−3^ and the deepest hole of −0.35 e^−^ Å^−3^ are found 0.97 and 0.53 Å from atoms C2 and Fe1, respectively.

For **2** the maximum residual peak of 0.38 e^−^ Å^−3^ and the deepest hole of −0.18 e^−^ Å^−3^ are found 0.81 and 0.39 Å from atoms H15 and C14, respectively.

For **3** the maximum residual peak of 0.58 e^−^ Å^−3^ and the deepest hole of −0.23 e^−^ Å^−3^ are found 1.15 and 1.25 Å from atoms C15 and K1, respectively. The peak is part of the minor component of disorder of the 18-crown-6 ring, which was not modeled (see above).

## Supplementary Material

Crystal structure: contains datablock(s) 1, 2, 3, global. DOI: 10.1107/S205698902101313X/yz2014sup1.cif

Structure factors: contains datablock(s) 1. DOI: 10.1107/S205698902101313X/yz20141sup2.hkl

Structure factors: contains datablock(s) 2. DOI: 10.1107/S205698902101313X/yz20142sup3.hkl

Structure factors: contains datablock(s) 3. DOI: 10.1107/S205698902101313X/yz20143sup4.hkl

CCDC references: 2127598, 2127597, 2127596

Additional supporting information:  crystallographic information; 3D view; checkCIF report

## Figures and Tables

**Table 1:** Selected geometric parameters (Å, °) for **1** [Chem scheme1]

Fe1—C33	1.800 (4)	C1—C2	1.415 (5)
Fe1—C24	1.840 (3)	C2—C3	1.399 (5)
Fe1—C15	1.847 (3)	C3—C4	1.421 (5)
Fe1—C3	2.034 (3)	C15—N1	1.167 (4)
Fe1—C2	2.044 (3)	C24—N2	1.165 (4)
Fe1—C4	2.125 (3)	C33—N3	1.181 (4)
Fe1—C1	2.172 (4)		
			
C33—Fe1—C24	90.40 (14)	C15—N1—C16	177.5 (4)
C33—Fe1—C15	94.97 (14)	C24—N2—C25	174.1 (3)
C24—Fe1—C15	100.38 (14)	C33—N3—C34	166.5 (3)

**Table 2:** Selected geometric parameters (Å, °) for **2** [Chem scheme1]

Fe1—C10	1.851 (3)	Fe1—C46	1.955 (2)
Fe1—C1	1.852 (2)	Fe1—C73	2.011 (2)
Fe1—C19	1.854 (3)	Fe1—C28	2.014 (2)
			
C10—Fe1—C1	84.67 (10)	C19—Fe1—C73	90.39 (10)
C10—Fe1—C19	94.05 (11)	C46—Fe1—C73	81.56 (9)
C1—Fe1—C19	177.81 (11)	C10—Fe1—C28	99.78 (10)
C10—Fe1—C46	171.45 (10)	C1—Fe1—C28	96.36 (10)
C1—Fe1—C46	86.80 (9)	C19—Fe1—C28	85.61 (10)
C19—Fe1—C46	94.46 (10)	C46—Fe1—C28	81.76 (10)
C10—Fe1—C73	97.51 (10)	C73—Fe1—C28	162.48 (9)
C1—Fe1—C73	88.02 (9)		

**Table 3:** Selected bond lengths (Å) for **3** [Chem scheme1]

K1—N1	2.772 (3)	K1—O1	2.835 (3)
K1—O5	2.797 (2)	K1—O2	2.846 (3)
K1—O4	2.831 (3)	K1—O6	2.959 (3)
K1—O3	2.832 (3)		

**Table 4:** Hydrogen-bond geometry (Å, °) for **2** [Chem scheme1]

*D*—H⋯*A*	*D*—H	H⋯*A*	*D*⋯*A*	*D*—H⋯*A*
N8—H8⋯N9	0.86	2.10	2.515 (13)	109
N8′—H8′⋯N9	0.86	2.22	2.644 (18)	110

**Table 5:** Experimental details

	**1**	**2**	**3**
Crystal data			
Chemical formula	[Fe(C_14_H_10_)(C_9_H_9_N)_3_]	[Fe(C_54_H_56_N_6_)(C_9_H_9_N)_3_]·2C_4_H_8_O	[K(C_9_H_8_N)(C_12_H_24_O_6_)]
*M*_r_	627.58	1382.62	433.57
Crystal system, space group	Monoclinic, *P*2_1_/*n*	Triclinic, *P*\overline{1}	Monoclinic, *P*2_1_/*n*
Temperature (K)	173	293	173
*a*, *b*, *c* (Å)	11.8528 (11), 10.9022 (10), 24.927 (2)	13.8912 (10), 15.4941 (11), 19.7902 (14)	10.784 (3), 9.754 (3), 21.783 (7)
α, β, γ (°)	90, 93.057 (2), 90	85.342 (3), 74.001 (3), 70.884 (3)	90, 91.864 (4), 90
*V* (Å^3^)	3216.5 (5)	3868.5 (5)	2290.1 (12)
*Z*	4	2	4
Radiation type	Mo *K*α	Mo *K*α	Mo *K*α
μ (mm^−1^)	0.50	0.25	0.27
Crystal size (mm)	0.23 × 0.14 × 0.08	0.34 × 0.30 × 0.24	0.24 × 0.18 × 0.15

Data collection			
Diffractometer	Siemens SMART CCD platform	Bruker SMART CCD platform	Bruker SMART CCD platform
Absorption correction	Multi-scan (*SADABS*; Krause *et al.*, 2015 ▸)	Multi-scan (*SADABS*; Krause *et al.*, 2015 ▸)	Multi-scan (*SADABS*; Krause *et al.*, 2015 ▸)
*T*_min_, *T*_max_	0.820, 1.000	0.925, 1.000	0.843, 1.000
No. of measured, independent and observed [*I* > 2σ(*I*)] reflections	24459, 5687, 4023	31905, 13640, 9367	21112, 4071, 2902
*R*_int_	0.087	0.040	0.062
(sin θ/λ)_max_ (Å^−1^)	0.596	0.596	0.596

Refinement			
*R*[*F* ^2^ > 2σ(*F* ^2^)], *wR*(*F* ^2^), *S*	0.060, 0.120, 1.05	0.051, 0.136, 1.02	0.059, 0.170, 1.05
No. of reflections	5687	13640	4071
No. of parameters	429	1192	355
No. of restraints	0	382	195
H-atom treatment	H atoms treated by a mixture of independent and constrained refinement	H atoms treated by a mixture of independent and constrained refinement	H-atom parameters constrained
Δρ_max_, Δρ_min_ (e Å^−3^)	0.36, −0.35	0.38, −0.18	0.58, −0.23

Computer programs: *SMART* and *SAINT* (Bruker, 2003 ▸), *SIR97* (Altomare *et al.*, 1999 ▸), *SHELXL2018/3* (Sheldrick, 2015 ▸), and *SHELXTL* (Sheldrick, 2008 ▸).

## supplementary crystallographic information

### [(1,2,3,4-η)-Anthracene]tris(2,6-dimethylphenyl isocyanide)iron (1) Crystal data

**Table:**

[Fe(C_14_H_10_)(C_9_H_9_N)_3_]	*F*(000) = 1320
*M**_r_* = 627.58	*D*_x_ = 1.296 Mg m^−^^3^
Monoclinic, *P*2_1_/*n*	Mo *K*α radiation, λ = 0.71073 Å
*a* = 11.8528 (11) Å	Cell parameters from 2566 reflections
*b* = 10.9022 (10) Å	θ = 2.5–24.3°
*c* = 24.927 (2) Å	µ = 0.50 mm^−^^1^
β = 93.057 (2)°	*T* = 173 K
*V* = 3216.5 (5) Å^3^	Block, orange
*Z* = 4	0.23 × 0.14 × 0.08 mm

### [(1,2,3,4-η)-Anthracene]tris(2,6-dimethylphenyl isocyanide)iron (1) Data collection

**Table:**

Siemens SMART CCD platform diffractometer	4023 reflections with *I* > 2σ(*I*)
Radiation source: normal-focus sealed tube	*R*_int_ = 0.087
ω scans	θ_max_ = 25.1°, θ_min_ = 1.6°
Absorption correction: multi-scan (SADABS; Krause *et al.*, 2015)	*h* = −14→13
*T*_min_ = 0.820, *T*_max_ = 1.000	*k* = −12→12
24459 measured reflections	*l* = −29→29
5687 independent reflections	

### [(1,2,3,4-η)-Anthracene]tris(2,6-dimethylphenyl isocyanide)iron (1) Refinement

**Table:**

Refinement on *F*^2^	Secondary atom site location: difference Fourier map
Least-squares matrix: full	Hydrogen site location: mixed
*R*[*F*^2^ > 2σ(*F*^2^)] = 0.060	H atoms treated by a mixture of independent and constrained refinement
*wR*(*F*^2^) = 0.120	*w* = 1/[σ^2^(*F*_o_^2^) + (0.0505*P*)^2^ + 1.1491*P*] where *P* = (*F*_o_^2^ + 2*F*_c_^2^)/3
*S* = 1.05	(Δ/σ)_max_ < 0.001
5687 reflections	Δρ_max_ = 0.36 e Å^−^^3^
429 parameters	Δρ_min_ = −0.35 e Å^−^^3^
0 restraints	Extinction correction: SHELXL-2018/3 (Sheldrick 2015), Fc^*^=kFc[1+0.001xFc^2^λ^3^/sin(2θ)]^-1/4^
Primary atom site location: structure-invariant direct methods	Extinction coefficient: 0.0108 (7)

### [(1,2,3,4-η)-Anthracene]tris(2,6-dimethylphenyl isocyanide)iron (1) Special details

**Table:**

Geometry. All esds (except the esd in the dihedral angle between two l.s. planes) are estimated using the full covariance matrix. The cell esds are taken into account individually in the estimation of esds in distances, angles and torsion angles; correlations between esds in cell parameters are only used when they are defined by crystal symmetry. An approximate (isotropic) treatment of cell esds is used for estimating esds involving l.s. planes.

### [(1,2,3,4-η)-Anthracene]tris(2,6-dimethylphenyl isocyanide)iron (1) Fractional atomic coordinates and isotropic or equivalent isotropic displacement parameters (Å^2^)

**Table:**

	*x*	*y*	*z*	*U*_iso_*/*U*_eq_	
Fe1	0.31214 (4)	0.23419 (4)	0.06206 (2)	0.02761 (16)	
C1	0.3597 (3)	0.1374 (3)	−0.00948 (15)	0.0344 (9)	
H1A	0.427 (3)	0.157 (3)	−0.0271 (14)	0.049 (11)*	
C2	0.3697 (3)	0.0671 (3)	0.03826 (16)	0.0378 (9)	
H2A	0.437 (3)	0.038 (3)	0.0535 (14)	0.040 (10)*	
C3	0.2706 (3)	0.0535 (3)	0.06579 (16)	0.0346 (9)	
H3A	0.267 (3)	0.007 (3)	0.0977 (15)	0.053 (12)*	
C4	0.1740 (3)	0.1151 (3)	0.04281 (14)	0.0286 (8)	
H4A	0.113 (3)	0.116 (3)	0.0631 (13)	0.032 (9)*	
C5	−0.0638 (3)	0.1476 (3)	−0.12786 (15)	0.0408 (9)	
H5A	−0.131036	0.146436	−0.108779	0.049*	
C6	−0.0713 (4)	0.1606 (3)	−0.18278 (16)	0.0502 (11)	
H6A	−0.143118	0.167987	−0.201270	0.060*	
C7	0.0268 (4)	0.1628 (3)	−0.21135 (16)	0.0549 (12)	
H7A	0.021654	0.171483	−0.249322	0.066*	
C8	0.1300 (4)	0.1527 (3)	−0.18487 (15)	0.0493 (11)	
H8A	0.195912	0.154133	−0.204906	0.059*	
C9	0.2481 (3)	0.1367 (3)	−0.09923 (15)	0.0381 (9)	
H9A	0.315408	0.138178	−0.118294	0.046*	
C10	0.0513 (3)	0.1235 (3)	−0.04268 (13)	0.0297 (8)	
H10A	−0.015070	0.117228	−0.023102	0.036*	
C11	0.2555 (3)	0.1312 (3)	−0.04438 (14)	0.0313 (8)	
C12	0.1552 (3)	0.1205 (3)	−0.01571 (13)	0.0276 (8)	
C13	0.0415 (3)	0.1359 (3)	−0.09917 (13)	0.0330 (8)	
C14	0.1416 (3)	0.1402 (3)	−0.12837 (14)	0.0348 (9)	
C15	0.2630 (3)	0.3741 (3)	0.02621 (14)	0.0301 (8)	
N1	0.2304 (2)	0.4627 (2)	0.00412 (12)	0.0356 (7)	
C16	0.1942 (3)	0.5721 (3)	−0.02091 (13)	0.0299 (8)	
C17	0.1030 (3)	0.5683 (3)	−0.05881 (13)	0.0323 (8)	
C18	0.0705 (3)	0.6776 (3)	−0.08270 (14)	0.0377 (9)	
H18A	0.008746	0.678274	−0.108631	0.045*	
C19	0.1257 (3)	0.7863 (3)	−0.06976 (14)	0.0389 (9)	
H19A	0.101896	0.860406	−0.086943	0.047*	
C20	0.2143 (3)	0.7875 (3)	−0.03237 (14)	0.0363 (8)	
H20A	0.251753	0.862681	−0.023879	0.044*	
C21	0.2506 (3)	0.6801 (3)	−0.00653 (13)	0.0320 (8)	
C22	0.0448 (3)	0.4489 (3)	−0.07298 (16)	0.0487 (10)	
H22A	−0.015053	0.463279	−0.100939	0.073*	
H22B	0.011639	0.414999	−0.040972	0.073*	
H22C	0.099809	0.390683	−0.086233	0.073*	
C23	0.3474 (3)	0.6811 (4)	0.03499 (17)	0.0537 (11)	
H23A	0.327591	0.632025	0.066055	0.081*	
H23B	0.363206	0.765629	0.046433	0.081*	
H23C	0.414650	0.646176	0.019532	0.081*	
C24	0.4511 (3)	0.2860 (3)	0.08974 (14)	0.0316 (8)	
N2	0.5361 (2)	0.3168 (3)	0.11121 (12)	0.0385 (7)	
C25	0.6315 (3)	0.3606 (3)	0.14059 (13)	0.0296 (8)	
C26	0.6539 (3)	0.4863 (3)	0.13917 (14)	0.0340 (8)	
C27	0.7473 (3)	0.5284 (3)	0.16943 (15)	0.0426 (9)	
H27A	0.764292	0.613569	0.169569	0.051*	
C28	0.8156 (3)	0.4497 (4)	0.19920 (15)	0.0469 (10)	
H28A	0.879716	0.480378	0.219426	0.056*	
C29	0.7912 (3)	0.3261 (4)	0.19977 (14)	0.0427 (9)	
H29A	0.838982	0.272388	0.220648	0.051*	
C30	0.6986 (3)	0.2783 (3)	0.17056 (14)	0.0348 (8)	
C31	0.5800 (3)	0.5698 (3)	0.10484 (17)	0.0539 (11)	
H31A	0.575586	0.539425	0.067785	0.081*	
H31B	0.612114	0.652662	0.105700	0.081*	
H31C	0.504147	0.571857	0.118604	0.081*	
C32	0.6720 (4)	0.1442 (3)	0.17089 (18)	0.0558 (12)	
H32A	0.731368	0.100511	0.192037	0.084*	
H32B	0.668099	0.113055	0.133959	0.084*	
H32C	0.599212	0.131257	0.186880	0.084*	
C33	0.2496 (3)	0.2732 (3)	0.12404 (14)	0.0316 (8)	
N3	0.2010 (2)	0.2945 (3)	0.16316 (12)	0.0386 (7)	
C34	0.1558 (3)	0.3463 (3)	0.20781 (14)	0.0322 (8)	
C35	0.1799 (3)	0.4689 (3)	0.21915 (15)	0.0415 (9)	
C36	0.1398 (3)	0.5169 (4)	0.26596 (18)	0.0538 (11)	
H36A	0.154918	0.600200	0.274916	0.065*	
C37	0.0787 (3)	0.4468 (4)	0.29957 (17)	0.0585 (13)	
H37A	0.054290	0.480930	0.332048	0.070*	
C38	0.0521 (3)	0.3257 (4)	0.28640 (16)	0.0496 (10)	
H38A	0.008566	0.278127	0.309637	0.060*	
C39	0.0888 (3)	0.2741 (3)	0.23960 (14)	0.0365 (8)	
C40	0.2463 (4)	0.5432 (4)	0.18151 (18)	0.0639 (13)	
H40B	0.251798	0.627969	0.194415	0.096*	
H40C	0.208241	0.541810	0.145624	0.096*	
H40D	0.322229	0.508409	0.179802	0.096*	
C41	0.0569 (3)	0.1459 (3)	0.22234 (17)	0.0484 (10)	
H41B	0.125365	0.099304	0.215470	0.073*	
H41C	0.007835	0.149243	0.189459	0.073*	
H41D	0.016726	0.105596	0.250836	0.073*	

### [(1,2,3,4-η)-Anthracene]tris(2,6-dimethylphenyl isocyanide)iron (1) Atomic displacement parameters (Å^2^)

**Table:**

	*U*^11^	*U*^22^	*U*^33^	*U*^12^	*U*^13^	*U*^23^
Fe1	0.0248 (3)	0.0193 (2)	0.0384 (3)	−0.0033 (2)	−0.0010 (2)	−0.0014 (2)
C1	0.030 (2)	0.0243 (17)	0.050 (2)	−0.0045 (15)	0.0110 (19)	−0.0073 (16)
C2	0.034 (2)	0.0200 (17)	0.058 (3)	0.0009 (15)	−0.005 (2)	−0.0062 (17)
C3	0.039 (2)	0.0187 (17)	0.046 (2)	−0.0078 (14)	−0.0027 (19)	0.0000 (16)
C4	0.028 (2)	0.0227 (17)	0.035 (2)	−0.0071 (14)	0.0047 (17)	0.0002 (14)
C5	0.050 (2)	0.0282 (19)	0.044 (2)	−0.0124 (16)	−0.003 (2)	0.0019 (16)
C6	0.071 (3)	0.036 (2)	0.042 (2)	−0.0143 (19)	−0.014 (2)	0.0046 (18)
C7	0.092 (4)	0.038 (2)	0.033 (2)	−0.020 (2)	−0.004 (3)	−0.0010 (18)
C8	0.076 (3)	0.033 (2)	0.041 (2)	−0.015 (2)	0.020 (2)	−0.0076 (17)
C9	0.043 (2)	0.0262 (18)	0.046 (2)	−0.0077 (16)	0.0167 (19)	−0.0104 (16)
C10	0.034 (2)	0.0229 (17)	0.0324 (19)	−0.0107 (14)	0.0061 (16)	−0.0002 (14)
C11	0.033 (2)	0.0197 (16)	0.042 (2)	−0.0054 (14)	0.0088 (17)	−0.0046 (14)
C12	0.032 (2)	0.0161 (15)	0.035 (2)	−0.0077 (13)	0.0047 (16)	−0.0042 (13)
C13	0.046 (2)	0.0202 (16)	0.033 (2)	−0.0109 (15)	0.0022 (18)	−0.0021 (14)
C14	0.054 (2)	0.0183 (16)	0.032 (2)	−0.0130 (15)	0.0089 (18)	−0.0047 (14)
C15	0.0269 (19)	0.0263 (18)	0.037 (2)	−0.0082 (14)	0.0034 (16)	−0.0060 (16)
N1	0.0382 (17)	0.0258 (16)	0.0432 (18)	0.0025 (13)	0.0062 (14)	0.0059 (13)
C16	0.0307 (19)	0.0235 (17)	0.036 (2)	0.0025 (14)	0.0096 (16)	0.0030 (14)
C17	0.0299 (19)	0.0338 (19)	0.034 (2)	−0.0008 (15)	0.0065 (16)	−0.0029 (15)
C18	0.033 (2)	0.045 (2)	0.035 (2)	0.0029 (17)	0.0002 (17)	−0.0013 (17)
C19	0.049 (2)	0.029 (2)	0.039 (2)	0.0106 (16)	0.0089 (19)	0.0046 (16)
C20	0.044 (2)	0.0275 (19)	0.037 (2)	−0.0039 (15)	0.0065 (18)	−0.0032 (15)
C21	0.032 (2)	0.0301 (18)	0.0338 (19)	−0.0009 (15)	0.0027 (16)	−0.0007 (15)
C22	0.050 (2)	0.042 (2)	0.054 (3)	−0.0151 (18)	0.000 (2)	−0.0075 (19)
C23	0.047 (2)	0.055 (2)	0.057 (3)	−0.011 (2)	−0.009 (2)	0.002 (2)
C24	0.0312 (19)	0.0214 (17)	0.043 (2)	0.0012 (14)	0.0041 (17)	−0.0005 (15)
N2	0.0294 (17)	0.0393 (17)	0.0463 (19)	−0.0059 (14)	−0.0011 (15)	−0.0091 (14)
C25	0.0219 (18)	0.0326 (18)	0.0344 (19)	−0.0039 (14)	0.0021 (15)	−0.0050 (15)
C26	0.035 (2)	0.0324 (19)	0.035 (2)	−0.0044 (15)	0.0035 (16)	−0.0024 (15)
C27	0.043 (2)	0.038 (2)	0.047 (2)	−0.0148 (18)	0.006 (2)	−0.0104 (18)
C28	0.033 (2)	0.069 (3)	0.038 (2)	−0.011 (2)	0.0007 (18)	−0.020 (2)
C29	0.033 (2)	0.060 (3)	0.035 (2)	0.0077 (18)	−0.0001 (18)	−0.0004 (18)
C30	0.0337 (19)	0.0333 (18)	0.038 (2)	0.0005 (15)	0.0071 (16)	−0.0003 (16)
C31	0.053 (3)	0.040 (2)	0.069 (3)	0.0018 (19)	−0.003 (2)	0.007 (2)
C32	0.058 (3)	0.035 (2)	0.074 (3)	0.0019 (19)	0.008 (2)	0.011 (2)
C33	0.0275 (18)	0.0254 (17)	0.041 (2)	−0.0044 (14)	−0.0090 (17)	−0.0011 (16)
N3	0.0342 (17)	0.0408 (18)	0.0401 (19)	−0.0038 (13)	−0.0034 (15)	−0.0076 (14)
C34	0.0280 (19)	0.0350 (19)	0.033 (2)	0.0043 (15)	−0.0060 (16)	−0.0070 (16)
C35	0.034 (2)	0.042 (2)	0.047 (2)	0.0007 (17)	−0.0158 (18)	−0.0085 (18)
C36	0.046 (3)	0.050 (2)	0.063 (3)	0.012 (2)	−0.018 (2)	−0.025 (2)
C37	0.042 (3)	0.085 (3)	0.047 (3)	0.027 (2)	−0.009 (2)	−0.027 (2)
C38	0.036 (2)	0.070 (3)	0.042 (2)	0.013 (2)	−0.0038 (19)	0.002 (2)
C39	0.0307 (19)	0.039 (2)	0.039 (2)	0.0108 (16)	−0.0059 (16)	0.0011 (17)
C40	0.068 (3)	0.049 (3)	0.073 (3)	−0.018 (2)	−0.017 (3)	0.005 (2)
C41	0.043 (2)	0.042 (2)	0.060 (3)	0.0004 (18)	0.000 (2)	0.0053 (19)

### [(1,2,3,4-η)-Anthracene]tris(2,6-dimethylphenyl isocyanide)iron (1) Geometric parameters (Å, º)

**Table:**

Fe1—C33	1.800 (4)	C21—C23	1.504 (5)
Fe1—C24	1.840 (3)	C22—H22A	0.9800
Fe1—C15	1.847 (3)	C22—H22B	0.9800
Fe1—C3	2.034 (3)	C22—H22C	0.9800
Fe1—C2	2.044 (3)	C23—H23A	0.9800
Fe1—C4	2.125 (3)	C23—H23B	0.9800
Fe1—C1	2.172 (4)	C23—H23C	0.9800
C1—C2	1.415 (5)	C24—N2	1.165 (4)
C1—C11	1.473 (5)	N2—C25	1.398 (4)
C1—H1A	0.96 (4)	C25—C30	1.389 (4)
C2—C3	1.399 (5)	C25—C26	1.396 (4)
C2—H2A	0.93 (3)	C26—C27	1.384 (5)
C3—C4	1.421 (5)	C26—C31	1.499 (5)
C3—H3A	0.94 (4)	C27—C28	1.371 (5)
C4—C12	1.465 (5)	C27—H27A	0.9500
C4—H4A	0.91 (3)	C28—C29	1.379 (5)
C5—C6	1.375 (5)	C28—H28A	0.9500
C5—C13	1.411 (5)	C29—C30	1.386 (5)
C5—H5A	0.9500	C29—H29A	0.9500
C6—C7	1.395 (6)	C30—C32	1.496 (5)
C6—H6A	0.9500	C31—H31A	0.9800
C7—C8	1.363 (6)	C31—H31B	0.9800
C7—H7A	0.9500	C31—H31C	0.9800
C8—C14	1.414 (5)	C32—H32A	0.9800
C8—H8A	0.9500	C32—H32B	0.9800
C9—C11	1.367 (5)	C32—H32C	0.9800
C9—C14	1.424 (5)	C33—N3	1.181 (4)
C9—H9A	0.9500	N3—C34	1.382 (4)
C10—C12	1.371 (4)	C34—C35	1.393 (5)
C10—C13	1.413 (5)	C34—C39	1.395 (5)
C10—H10A	0.9500	C35—C36	1.386 (6)
C11—C12	1.425 (4)	C35—C40	1.494 (6)
C13—C14	1.425 (5)	C36—C37	1.369 (6)
C15—N1	1.167 (4)	C36—H36A	0.9500
N1—C16	1.403 (4)	C37—C38	1.393 (6)
C16—C21	1.392 (4)	C37—H37A	0.9500
C16—C17	1.398 (5)	C38—C39	1.386 (5)
C17—C18	1.378 (5)	C38—H38A	0.9500
C17—C22	1.507 (4)	C39—C41	1.505 (5)
C18—C19	1.384 (5)	C40—H40B	0.9800
C18—H18A	0.9500	C40—H40C	0.9800
C19—C20	1.367 (5)	C40—H40D	0.9800
C19—H19A	0.9500	C41—H41B	0.9800
C20—C21	1.393 (4)	C41—H41C	0.9800
C20—H20A	0.9500	C41—H41D	0.9800
			
C33—Fe1—C24	90.40 (14)	C20—C19—C18	120.3 (3)
C33—Fe1—C15	94.97 (14)	C20—C19—H19A	119.9
C24—Fe1—C15	100.38 (14)	C18—C19—H19A	119.9
C33—Fe1—C3	94.45 (15)	C19—C20—C21	120.9 (3)
C24—Fe1—C3	119.62 (14)	C19—C20—H20A	119.5
C15—Fe1—C3	138.74 (14)	C21—C20—H20A	119.5
C33—Fe1—C2	128.22 (15)	C16—C21—C20	117.4 (3)
C24—Fe1—C2	94.40 (14)	C16—C21—C23	121.4 (3)
C15—Fe1—C2	134.22 (15)	C20—C21—C23	121.2 (3)
C3—Fe1—C2	40.14 (14)	C17—C22—H22A	109.5
C33—Fe1—C4	89.44 (14)	C17—C22—H22B	109.5
C24—Fe1—C4	159.38 (13)	H22A—C22—H22B	109.5
C15—Fe1—C4	100.17 (13)	C17—C22—H22C	109.5
C3—Fe1—C4	39.90 (13)	H22A—C22—H22C	109.5
C2—Fe1—C4	69.82 (14)	H22B—C22—H22C	109.5
C33—Fe1—C1	162.98 (14)	C21—C23—H23A	109.5
C24—Fe1—C1	101.02 (14)	C21—C23—H23B	109.5
C15—Fe1—C1	95.33 (14)	H23A—C23—H23B	109.5
C3—Fe1—C1	68.97 (14)	C21—C23—H23C	109.5
C2—Fe1—C1	39.09 (14)	H23A—C23—H23C	109.5
C4—Fe1—C1	75.42 (14)	H23B—C23—H23C	109.5
C2—C1—C11	120.1 (3)	N2—C24—Fe1	174.7 (3)
C2—C1—Fe1	65.6 (2)	C24—N2—C25	174.1 (3)
C11—C1—Fe1	105.2 (2)	C30—C25—C26	122.9 (3)
C2—C1—H1A	118 (2)	C30—C25—N2	119.0 (3)
C11—C1—H1A	116 (2)	C26—C25—N2	118.1 (3)
Fe1—C1—H1A	122 (2)	C27—C26—C25	117.4 (3)
C3—C2—C1	115.8 (3)	C27—C26—C31	122.4 (3)
C3—C2—Fe1	69.5 (2)	C25—C26—C31	120.3 (3)
C1—C2—Fe1	75.3 (2)	C28—C27—C26	121.3 (3)
C3—C2—H2A	120 (2)	C28—C27—H27A	119.3
C1—C2—H2A	124 (2)	C26—C27—H27A	119.3
Fe1—C2—H2A	119 (2)	C27—C28—C29	120.0 (3)
C2—C3—C4	115.6 (3)	C27—C28—H28A	120.0
C2—C3—Fe1	70.33 (19)	C29—C28—H28A	120.0
C4—C3—Fe1	73.50 (19)	C28—C29—C30	121.5 (3)
C2—C3—H3A	123 (2)	C28—C29—H29A	119.3
C4—C3—H3A	121 (2)	C30—C29—H29A	119.3
Fe1—C3—H3A	125 (2)	C29—C30—C25	117.0 (3)
C3—C4—C12	119.8 (3)	C29—C30—C32	121.7 (3)
C3—C4—Fe1	66.60 (18)	C25—C30—C32	121.3 (3)
C12—C4—Fe1	106.0 (2)	C26—C31—H31A	109.5
C3—C4—H4A	116 (2)	C26—C31—H31B	109.5
C12—C4—H4A	118 (2)	H31A—C31—H31B	109.5
Fe1—C4—H4A	120 (2)	C26—C31—H31C	109.5
C6—C5—C13	121.5 (4)	H31A—C31—H31C	109.5
C6—C5—H5A	119.2	H31B—C31—H31C	109.5
C13—C5—H5A	119.2	C30—C32—H32A	109.5
C5—C6—C7	119.9 (4)	C30—C32—H32B	109.5
C5—C6—H6A	120.0	H32A—C32—H32B	109.5
C7—C6—H6A	120.0	C30—C32—H32C	109.5
C8—C7—C6	120.1 (4)	H32A—C32—H32C	109.5
C8—C7—H7A	119.9	H32B—C32—H32C	109.5
C6—C7—H7A	119.9	N3—C33—Fe1	174.9 (3)
C7—C8—C14	121.8 (4)	C33—N3—C34	166.5 (3)
C7—C8—H8A	119.1	N3—C34—C35	118.1 (3)
C14—C8—H8A	119.1	N3—C34—C39	118.9 (3)
C11—C9—C14	121.3 (3)	C35—C34—C39	122.9 (3)
C11—C9—H9A	119.3	C36—C35—C34	117.2 (4)
C14—C9—H9A	119.3	C36—C35—C40	122.7 (4)
C12—C10—C13	121.0 (3)	C34—C35—C40	120.1 (4)
C12—C10—H10A	119.5	C37—C36—C35	121.4 (4)
C13—C10—H10A	119.5	C37—C36—H36A	119.3
C9—C11—C12	119.6 (3)	C35—C36—H36A	119.3
C9—C11—C1	126.6 (3)	C36—C37—C38	120.4 (4)
C12—C11—C1	113.8 (3)	C36—C37—H37A	119.8
C10—C12—C11	120.3 (3)	C38—C37—H37A	119.8
C10—C12—C4	125.0 (3)	C39—C38—C37	120.4 (4)
C11—C12—C4	114.6 (3)	C39—C38—H38A	119.8
C5—C13—C10	122.5 (3)	C37—C38—H38A	119.8
C5—C13—C14	118.4 (3)	C38—C39—C34	117.5 (3)
C10—C13—C14	119.1 (3)	C38—C39—C41	122.2 (4)
C8—C14—C9	123.2 (4)	C34—C39—C41	120.3 (3)
C8—C14—C13	118.2 (4)	C35—C40—H40B	109.5
C9—C14—C13	118.6 (3)	C35—C40—H40C	109.5
N1—C15—Fe1	178.8 (3)	H40B—C40—H40C	109.5
C15—N1—C16	177.5 (4)	C35—C40—H40D	109.5
C21—C16—C17	122.9 (3)	H40B—C40—H40D	109.5
C21—C16—N1	118.3 (3)	H40C—C40—H40D	109.5
C17—C16—N1	118.9 (3)	C39—C41—H41B	109.5
C18—C17—C16	117.1 (3)	C39—C41—H41C	109.5
C18—C17—C22	122.1 (3)	H41B—C41—H41C	109.5
C16—C17—C22	120.8 (3)	C39—C41—H41D	109.5
C17—C18—C19	121.5 (3)	H41B—C41—H41D	109.5
C17—C18—H18A	119.3	H41C—C41—H41D	109.5
C19—C18—H18A	119.3		
			
C11—C1—C2—C3	−35.8 (5)	C21—C16—C17—C22	−180.0 (3)
Fe1—C1—C2—C3	58.2 (3)	N1—C16—C17—C22	−0.4 (5)
C11—C1—C2—Fe1	−93.9 (3)	C16—C17—C18—C19	0.0 (5)
C1—C2—C3—C4	−1.7 (4)	C22—C17—C18—C19	−179.2 (3)
Fe1—C2—C3—C4	59.6 (3)	C17—C18—C19—C20	−0.3 (5)
C1—C2—C3—Fe1	−61.3 (3)	C18—C19—C20—C21	−0.2 (5)
C2—C3—C4—C12	37.7 (4)	C17—C16—C21—C20	−1.3 (5)
Fe1—C3—C4—C12	95.6 (3)	N1—C16—C21—C20	179.1 (3)
C2—C3—C4—Fe1	−57.9 (3)	C17—C16—C21—C23	179.2 (3)
C13—C5—C6—C7	−0.2 (5)	N1—C16—C21—C23	−0.4 (5)
C5—C6—C7—C8	−0.3 (5)	C19—C20—C21—C16	0.9 (5)
C6—C7—C8—C14	−0.2 (5)	C19—C20—C21—C23	−179.5 (3)
C14—C9—C11—C12	4.3 (5)	C30—C25—C26—C27	−0.2 (5)
C14—C9—C11—C1	−174.6 (3)	N2—C25—C26—C27	178.6 (3)
C2—C1—C11—C9	−144.3 (4)	C30—C25—C26—C31	178.4 (3)
Fe1—C1—C11—C9	145.5 (3)	N2—C25—C26—C31	−2.8 (5)
C2—C1—C11—C12	36.8 (4)	C25—C26—C27—C28	0.6 (5)
Fe1—C1—C11—C12	−33.4 (3)	C31—C26—C27—C28	−177.9 (4)
C13—C10—C12—C11	0.7 (4)	C26—C27—C28—C29	−0.6 (6)
C13—C10—C12—C4	176.3 (3)	C27—C28—C29—C30	0.2 (6)
C9—C11—C12—C10	−4.2 (4)	C28—C29—C30—C25	0.1 (5)
C1—C11—C12—C10	174.8 (3)	C28—C29—C30—C32	179.8 (3)
C9—C11—C12—C4	179.8 (3)	C26—C25—C30—C29	−0.2 (5)
C1—C11—C12—C4	−1.2 (4)	N2—C25—C30—C29	−179.0 (3)
C3—C4—C12—C10	148.6 (3)	C26—C25—C30—C32	−179.8 (3)
Fe1—C4—C12—C10	−139.5 (3)	N2—C25—C30—C32	1.4 (5)
C3—C4—C12—C11	−35.6 (4)	C33—N3—C34—C35	2.0 (15)
Fe1—C4—C12—C11	36.2 (3)	C33—N3—C34—C39	−177.6 (13)
C6—C5—C13—C10	179.4 (3)	N3—C34—C35—C36	−176.0 (3)
C6—C5—C13—C14	1.2 (5)	C39—C34—C35—C36	3.6 (5)
C12—C10—C13—C5	−175.7 (3)	N3—C34—C35—C40	4.2 (5)
C12—C10—C13—C14	2.6 (4)	C39—C34—C35—C40	−176.2 (3)
C7—C8—C14—C9	−176.2 (3)	C34—C35—C36—C37	−0.1 (5)
C7—C8—C14—C13	1.2 (5)	C40—C35—C36—C37	179.7 (4)
C11—C9—C14—C8	176.3 (3)	C35—C36—C37—C38	−2.2 (6)
C11—C9—C14—C13	−1.0 (5)	C36—C37—C38—C39	1.1 (6)
C5—C13—C14—C8	−1.6 (4)	C37—C38—C39—C34	2.2 (5)
C10—C13—C14—C8	−179.9 (3)	C37—C38—C39—C41	−176.7 (3)
C5—C13—C14—C9	175.8 (3)	N3—C34—C39—C38	175.0 (3)
C10—C13—C14—C9	−2.4 (4)	C35—C34—C39—C38	−4.6 (5)
C21—C16—C17—C18	0.8 (5)	N3—C34—C39—C41	−6.2 (5)
N1—C16—C17—C18	−179.6 (3)	C35—C34—C39—C41	174.2 (3)

### {5,6-Bis(2,6-dimethylanilino)-3-(2,6-dimethylphenyl)-1,2,7-tris[(2,6-dimethylphenyl)imino]-3-azoniahept-3-ene-1,4,7-triido}tris(2,6-dimethylphenyl isocyanide)iron tetrahydrofuran disolvate (2) Crystal data

**Table:**

[Fe(C_54_H_56_N_6_)(C_9_H_9_N)_3_]·2C_4_H_8_O	*Z* = 2
*M**_r_* = 1382.62	*F*(000) = 1476
Triclinic, *P*1	*D*_x_ = 1.187 Mg m^−^^3^
*a* = 13.8912 (10) Å	Mo *K*α radiation, λ = 0.71073 Å
*b* = 15.4941 (11) Å	Cell parameters from 3451 reflections
*c* = 19.7902 (14) Å	θ = 2.3–22.7°
α = 85.342 (3)°	µ = 0.25 mm^−^^1^
β = 74.001 (3)°	*T* = 293 K
γ = 70.884 (3)°	Block, dark red
*V* = 3868.5 (5) Å^3^	0.34 × 0.30 × 0.24 mm

### {5,6-Bis(2,6-dimethylanilino)-3-(2,6-dimethylphenyl)-1,2,7-tris[(2,6-dimethylphenyl)imino]-3-azoniahept-3-ene-1,4,7-triido}tris(2,6-dimethylphenyl isocyanide)iron tetrahydrofuran disolvate (2) Data collection

**Table:**

Bruker SMART CCD platform diffractometer	9367 reflections with *I* > 2σ(*I*)
Radiation source: fine-focus sealed tube	*R*_int_ = 0.040
ω scans	θ_max_ = 25.1°, θ_min_ = 1.6°
Absorption correction: multi-scan (SADABS; Krause *et al.*, 2015)	*h* = −16→16
*T*_min_ = 0.925, *T*_max_ = 1.000	*k* = −18→18
31905 measured reflections	*l* = −23→23
13640 independent reflections	

### {5,6-Bis(2,6-dimethylanilino)-3-(2,6-dimethylphenyl)-1,2,7-tris[(2,6-dimethylphenyl)imino]-3-azoniahept-3-ene-1,4,7-triido}tris(2,6-dimethylphenyl isocyanide)iron tetrahydrofuran disolvate (2) Refinement

**Table:**

Refinement on *F*^2^	Primary atom site location: structure-invariant direct methods
Least-squares matrix: full	Secondary atom site location: difference Fourier map
*R*[*F*^2^ > 2σ(*F*^2^)] = 0.051	Hydrogen site location: mixed
*wR*(*F*^2^) = 0.136	H atoms treated by a mixture of independent and constrained refinement
*S* = 1.02	*w* = 1/[σ^2^(*F*_o_^2^) + (0.060*P*)^2^ + 0.9501*P*] where *P* = (*F*_o_^2^ + 2*F*_c_^2^)/3
13640 reflections	(Δ/σ)_max_ < 0.001
1192 parameters	Δρ_max_ = 0.38 e Å^−^^3^
382 restraints	Δρ_min_ = −0.18 e Å^−^^3^

### {5,6-Bis(2,6-dimethylanilino)-3-(2,6-dimethylphenyl)-1,2,7-tris[(2,6-dimethylphenyl)imino]-3-azoniahept-3-ene-1,4,7-triido}tris(2,6-dimethylphenyl isocyanide)iron tetrahydrofuran disolvate (2) Special details

**Table:**

Geometry. All esds (except the esd in the dihedral angle between two l.s. planes) are estimated using the full covariance matrix. The cell esds are taken into account individually in the estimation of esds in distances, angles and torsion angles; correlations between esds in cell parameters are only used when they are defined by crystal symmetry. An approximate (isotropic) treatment of cell esds is used for estimating esds involving l.s. planes.
Refinement. Two CNXyl groups were modeled as disordered over two positions each: N1/C2-C9, 0.52 (2):0.48 (2) and N8/C65-C72, 0.57 (2):0.43 (2). The two thf solvent molecules were modeled as disordered over two positions each: O1/C82-C85, 0.55 (2):0.45 (2) and O2/C86-C89, 0.69 (1):0.31 (1).For the various pairs of components of disorder, analogous bond lengths and angles were restrained to be similar and anisotropic displacement parameters for proximal atoms were restrained to be similar. Bond lengths for the thf solvent molecules were restrained toward ideal values. Anisotropic displacement parameters for the thf solvent molecules were also restrained toward the expected motion relative to bond direction.

### {5,6-Bis(2,6-dimethylanilino)-3-(2,6-dimethylphenyl)-1,2,7-tris[(2,6-dimethylphenyl)imino]-3-azoniahept-3-ene-1,4,7-triido}tris(2,6-dimethylphenyl isocyanide)iron tetrahydrofuran disolvate (2) Fractional atomic coordinates and isotropic or equivalent isotropic displacement parameters (Å^2^)

**Table:**

	*x*	*y*	*z*	*U*_iso_*/*U*_eq_	Occ. (<1)
Fe1	0.35113 (3)	0.21957 (2)	0.30062 (2)	0.03572 (11)	
C1	0.25165 (18)	0.18309 (16)	0.36939 (12)	0.0374 (5)	
N1	0.1973 (17)	0.163 (2)	0.4210 (8)	0.038 (3)	0.48 (2)
C2	0.1256 (12)	0.1360 (14)	0.4769 (8)	0.040 (3)	0.48 (2)
C3	0.1659 (12)	0.0698 (12)	0.5224 (9)	0.050 (3)	0.48 (2)
C4	0.0935 (14)	0.0492 (11)	0.5792 (8)	0.084 (4)	0.48 (2)
H4	0.117586	0.005216	0.610856	0.101*	0.48 (2)
C5	−0.0125 (14)	0.0917 (13)	0.5900 (10)	0.114 (5)	0.48 (2)
H5	−0.059463	0.077798	0.629450	0.137*	0.48 (2)
C6	−0.0499 (12)	0.1545 (12)	0.5433 (9)	0.088 (4)	0.48 (2)
H6	−0.122488	0.181145	0.550534	0.106*	0.48 (2)
C7	0.0172 (12)	0.1794 (11)	0.4858 (8)	0.053 (3)	0.48 (2)
C8	0.2805 (13)	0.017 (2)	0.5158 (18)	0.069 (5)	0.48 (2)
H8A	0.286422	−0.042250	0.535705	0.103*	0.48 (2)
H8B	0.309270	0.049390	0.540343	0.103*	0.48 (2)
H8C	0.318798	0.011109	0.467017	0.103*	0.48 (2)
C9	−0.035 (2)	0.2440 (19)	0.4353 (16)	0.089 (6)	0.48 (2)
H9A	−0.054436	0.210192	0.405471	0.133*	0.48 (2)
H9B	0.012745	0.273556	0.407097	0.133*	0.48 (2)
H9C	−0.097647	0.289155	0.461464	0.133*	0.48 (2)
N1'	0.1885 (16)	0.1561 (19)	0.4099 (8)	0.039 (3)	0.52 (2)
C2'	0.1201 (11)	0.1246 (12)	0.4650 (8)	0.040 (3)	0.52 (2)
C3'	0.1615 (12)	0.0563 (12)	0.5086 (8)	0.050 (3)	0.52 (2)
C4'	0.0903 (12)	0.0241 (10)	0.5586 (8)	0.078 (3)	0.52 (2)
H4'	0.115114	−0.021889	0.588672	0.093*	0.52 (2)
C5'	−0.0159 (11)	0.0583 (12)	0.5648 (8)	0.099 (4)	0.52 (2)
H5'	−0.062225	0.034129	0.597690	0.119*	0.52 (2)
C6'	−0.0538 (11)	0.1280 (11)	0.5227 (8)	0.082 (4)	0.52 (2)
H6'	−0.126460	0.152483	0.528949	0.098*	0.52 (2)
C7'	0.0115 (11)	0.1629 (10)	0.4718 (7)	0.052 (3)	0.52 (2)
C8'	0.2783 (13)	0.024 (2)	0.5028 (18)	0.074 (5)	0.52 (2)
H8D	0.313260	0.054203	0.463980	0.111*	0.52 (2)
H8E	0.306287	−0.040505	0.495216	0.111*	0.52 (2)
H8F	0.289881	0.039270	0.545434	0.111*	0.52 (2)
C9'	−0.024 (2)	0.2447 (16)	0.4271 (14)	0.081 (5)	0.52 (2)
H9D	0.026936	0.238549	0.382300	0.121*	0.52 (2)
H9E	−0.031125	0.299351	0.450271	0.121*	0.52 (2)
H9F	−0.091234	0.248324	0.420245	0.121*	0.52 (2)
C10	0.34098 (19)	0.29901 (17)	0.36912 (13)	0.0430 (6)	
N2	0.32553 (18)	0.34287 (15)	0.41886 (12)	0.0532 (6)	
C11	0.3071 (2)	0.39771 (19)	0.47604 (16)	0.0601 (8)	
C12	0.3699 (3)	0.4569 (2)	0.4668 (2)	0.0751 (10)	
C13	0.3503 (4)	0.5122 (3)	0.5228 (3)	0.1084 (15)	
H13	0.389612	0.551578	0.519289	0.130*	
C14	0.2763 (4)	0.5113 (3)	0.5825 (3)	0.1104 (17)	
H14	0.265382	0.550597	0.618980	0.133*	
C15	0.2149 (3)	0.4537 (3)	0.5921 (2)	0.1005 (14)	
H15	0.164033	0.454590	0.634154	0.121*	
C16	0.2314 (3)	0.3935 (2)	0.53630 (17)	0.0723 (9)	
C17	0.4513 (3)	0.4572 (3)	0.4001 (2)	0.0970 (12)	
H17A	0.419412	0.468432	0.361580	0.146*	
H17B	0.505805	0.398944	0.393440	0.146*	
H17C	0.481311	0.504254	0.402151	0.146*	
C18	0.1678 (3)	0.3303 (3)	0.54419 (18)	0.0888 (11)	
H18A	0.110799	0.345926	0.586198	0.133*	
H18B	0.212062	0.268604	0.547200	0.133*	
H18C	0.139776	0.335815	0.504237	0.133*	
C19	0.4540 (2)	0.25518 (16)	0.23412 (13)	0.0433 (6)	
N3	0.51689 (18)	0.27967 (16)	0.19195 (12)	0.0577 (6)	
C20	0.5842 (2)	0.3167 (2)	0.13985 (18)	0.0667 (8)	
C21	0.5967 (3)	0.2979 (3)	0.0692 (2)	0.0917 (12)	
C22	0.6598 (5)	0.3372 (4)	0.0190 (3)	0.141 (2)	
H22	0.669592	0.326608	−0.028387	0.169*	
C23	0.7074 (5)	0.3904 (5)	0.0376 (4)	0.158 (2)	
H23	0.749511	0.415784	0.002305	0.190*	
C24	0.6971 (4)	0.4091 (4)	0.1052 (3)	0.1276 (18)	
H24	0.732090	0.445884	0.115935	0.153*	
C25	0.6318 (3)	0.3716 (3)	0.1599 (2)	0.0947 (12)	
C26	0.5409 (4)	0.2409 (3)	0.0490 (2)	0.1274 (17)	
H26A	0.468143	0.260308	0.075514	0.191*	
H26B	0.573494	0.177966	0.058805	0.191*	
H26C	0.545128	0.247637	−0.000267	0.191*	
C27	0.6145 (4)	0.3914 (3)	0.2352 (3)	0.1335 (18)	
H27A	0.640446	0.440613	0.239048	0.200*	
H27B	0.651428	0.337972	0.256977	0.200*	
H27C	0.540285	0.408553	0.258234	0.200*	
C28	0.24879 (19)	0.30335 (17)	0.25034 (12)	0.0406 (6)	
N4	0.20694 (17)	0.38993 (14)	0.24343 (12)	0.0509 (5)	
C29	0.2172 (2)	0.45872 (17)	0.28145 (16)	0.0539 (7)	
C30	0.2816 (2)	0.51023 (19)	0.24651 (18)	0.0640 (8)	
C31	0.2848 (3)	0.5822 (2)	0.2823 (2)	0.0820 (10)	
H31	0.328483	0.616191	0.259955	0.098*	
C32	0.2248 (3)	0.6042 (2)	0.3502 (2)	0.0910 (12)	
H32	0.229517	0.651535	0.373849	0.109*	
C33	0.1577 (3)	0.5562 (2)	0.38299 (19)	0.0775 (10)	
H33	0.115221	0.572878	0.428293	0.093*	
C34	0.1524 (2)	0.48277 (19)	0.34933 (17)	0.0608 (8)	
C35	0.3405 (3)	0.4929 (2)	0.17021 (19)	0.0876 (11)	
H35A	0.377563	0.428753	0.162594	0.131*	
H35B	0.390138	0.526179	0.157491	0.131*	
H35C	0.291318	0.512636	0.141852	0.131*	
C36	0.0763 (3)	0.4330 (2)	0.38418 (18)	0.0772 (9)	
H36A	0.114516	0.370046	0.389743	0.116*	
H36B	0.029164	0.437033	0.355702	0.116*	
H36C	0.036362	0.460045	0.429484	0.116*	
C37	0.21966 (19)	0.25081 (16)	0.20101 (12)	0.0395 (6)	
N5	0.15384 (17)	0.27524 (14)	0.16458 (11)	0.0496 (5)	
C38	0.0871 (2)	0.36446 (18)	0.15718 (15)	0.0548 (7)	
C39	−0.0167 (3)	0.3937 (2)	0.19960 (18)	0.0687 (9)	
C40	−0.0847 (3)	0.4763 (3)	0.1850 (3)	0.0979 (12)	
H40	−0.153475	0.496925	0.213467	0.118*	
C41	−0.0532 (4)	0.5280 (3)	0.1299 (3)	0.1214 (17)	
H41	−0.099957	0.583743	0.121359	0.146*	
C42	0.0480 (4)	0.4979 (3)	0.0867 (2)	0.1056 (14)	
H42	0.068513	0.532855	0.048386	0.127*	
C43	0.1197 (3)	0.4162 (2)	0.09944 (17)	0.0698 (9)	
C44	−0.0534 (3)	0.3352 (3)	0.2585 (2)	0.0938 (11)	
H44A	−0.040509	0.275608	0.240580	0.141*	
H44B	−0.127639	0.362572	0.279375	0.141*	
H44C	−0.015496	0.330053	0.293340	0.141*	
C45	0.2297 (3)	0.3817 (3)	0.05277 (19)	0.0975 (12)	
H45A	0.244439	0.319767	0.038922	0.146*	
H45B	0.279026	0.384155	0.077726	0.146*	
H45C	0.236145	0.419151	0.011719	0.146*	
C46	0.34745 (17)	0.12871 (16)	0.23919 (11)	0.0358 (5)	
N6	0.27881 (15)	0.15579 (13)	0.19868 (10)	0.0387 (5)	
C47	0.2492 (2)	0.09565 (17)	0.16096 (14)	0.0475 (6)	
C48	0.1703 (2)	0.05989 (19)	0.19782 (17)	0.0618 (8)	
C49	0.1442 (3)	0.0007 (3)	0.1624 (3)	0.0936 (12)	
H49	0.092437	−0.024861	0.186113	0.112*	
C50	0.1938 (4)	−0.0203 (3)	0.0931 (3)	0.1080 (15)	
H50	0.175252	−0.060106	0.070233	0.130*	
C51	0.2700 (4)	0.0162 (2)	0.0569 (2)	0.0883 (12)	
H51	0.302034	0.001541	0.009619	0.106*	
C52	0.3005 (3)	0.07522 (19)	0.08996 (15)	0.0597 (8)	
C53	0.1133 (3)	0.0839 (2)	0.27325 (19)	0.0793 (10)	
H53A	0.090769	0.148977	0.279523	0.119*	
H53B	0.159759	0.055074	0.302332	0.119*	
H53C	0.052766	0.063136	0.286284	0.119*	
C54	0.3860 (3)	0.1129 (2)	0.05188 (16)	0.0798 (10)	
H54A	0.362848	0.177606	0.060169	0.120*	
H54B	0.402603	0.101166	0.002415	0.120*	
H54C	0.447690	0.084315	0.068461	0.120*	
C55	0.40919 (18)	0.03657 (16)	0.24366 (12)	0.0376 (5)	
N7	0.42428 (19)	−0.03613 (14)	0.19870 (11)	0.0469 (5)	
H7	0.368 (2)	−0.0414 (17)	0.1943 (13)	0.047 (8)*	
C56	0.5200 (2)	−0.10162 (18)	0.16579 (13)	0.0517 (7)	
C57	0.5144 (3)	−0.1858 (2)	0.14824 (15)	0.0666 (9)	
C58	0.6081 (4)	−0.2537 (2)	0.11867 (18)	0.0911 (12)	
H58	0.605816	−0.309865	0.107167	0.109*	
C59	0.7037 (4)	−0.2398 (3)	0.1061 (2)	0.1055 (16)	
H59	0.765816	−0.287274	0.088764	0.127*	
C60	0.7078 (3)	−0.1557 (3)	0.11908 (17)	0.0909 (12)	
H60	0.773083	−0.146271	0.108416	0.109*	
C61	0.6169 (2)	−0.0840 (2)	0.14781 (14)	0.0623 (8)	
C62	0.4103 (3)	−0.2018 (2)	0.15989 (18)	0.0833 (11)	
H62A	0.371305	−0.189685	0.208266	0.125*	
H62B	0.422128	−0.264150	0.148473	0.125*	
H62C	0.370665	−0.161861	0.130402	0.125*	
C63	0.6280 (2)	0.0076 (2)	0.15438 (16)	0.0756 (9)	
H63A	0.572458	0.054107	0.139812	0.113*	
H63B	0.695168	0.009104	0.125112	0.113*	
H63C	0.623362	0.018380	0.202434	0.113*	
C64	0.45758 (17)	0.02443 (16)	0.29812 (11)	0.0366 (5)	
N8	0.5048 (13)	−0.0503 (6)	0.3313 (10)	0.041 (2)	0.568 (16)
H8	0.529233	−0.039870	0.364177	0.049*	0.568 (16)
C65	0.5203 (11)	−0.1449 (8)	0.320 (2)	0.043 (2)	0.568 (16)
C66	0.4371 (11)	−0.1796 (9)	0.3306 (13)	0.054 (3)	0.568 (16)
C67	0.4577 (10)	−0.2743 (8)	0.3258 (10)	0.068 (3)	0.568 (16)
H67	0.402487	−0.298439	0.333925	0.081*	0.568 (16)
C68	0.5607 (12)	−0.3305 (7)	0.3090 (6)	0.076 (4)	0.568 (16)
H68	0.575015	−0.392896	0.303746	0.091*	0.568 (16)
C69	0.6438 (11)	−0.2959 (8)	0.2996 (8)	0.075 (3)	0.568 (16)
H69	0.712835	−0.335344	0.287442	0.090*	0.568 (16)
C70	0.6254 (11)	−0.2029 (8)	0.3080 (13)	0.055 (3)	0.568 (16)
C71	0.3243 (11)	−0.1204 (11)	0.3543 (10)	0.065 (3)	0.568 (16)
H71A	0.279197	−0.151494	0.345844	0.098*	0.568 (16)
H71B	0.314728	−0.064218	0.328714	0.098*	0.568 (16)
H71C	0.306562	−0.107421	0.403678	0.098*	0.568 (16)
C72	0.7172 (13)	−0.1687 (14)	0.2994 (12)	0.081 (4)	0.568 (16)
H72A	0.702402	−0.127201	0.337189	0.121*	0.568 (16)
H72B	0.728860	−0.137668	0.255390	0.121*	0.568 (16)
H72C	0.779292	−0.219388	0.300150	0.121*	0.568 (16)
N8'	0.5095 (16)	−0.0569 (8)	0.3213 (14)	0.042 (4)	0.432 (16)
H8'	0.556817	−0.053030	0.340151	0.050*	0.432 (16)
C65'	0.5056 (15)	−0.1479 (11)	0.322 (3)	0.051 (3)	0.432 (16)
C66'	0.4096 (14)	−0.1637 (10)	0.3363 (17)	0.052 (3)	0.432 (16)
C67'	0.4144 (14)	−0.2542 (11)	0.3297 (14)	0.074 (4)	0.432 (16)
H67'	0.352638	−0.267872	0.333863	0.089*	0.432 (16)
C68'	0.5091 (15)	−0.3227 (11)	0.3172 (9)	0.078 (4)	0.432 (16)
H68'	0.509234	−0.382652	0.315929	0.094*	0.432 (16)
C69'	0.6055 (13)	−0.3071 (11)	0.3063 (10)	0.073 (4)	0.432 (16)
H69'	0.668441	−0.355385	0.298997	0.088*	0.432 (16)
C70'	0.6047 (15)	−0.2166 (12)	0.3066 (19)	0.054 (3)	0.432 (16)
C71'	0.3032 (15)	−0.0927 (15)	0.3537 (14)	0.070 (4)	0.432 (16)
H71D	0.257377	−0.112164	0.393280	0.105*	0.432 (16)
H71E	0.274080	−0.084125	0.314022	0.105*	0.432 (16)
H71F	0.309763	−0.036180	0.365077	0.105*	0.432 (16)
C72'	0.7029 (18)	−0.1920 (18)	0.2990 (14)	0.070 (4)	0.432 (16)
H72D	0.696102	−0.134069	0.276136	0.105*	0.432 (16)
H72E	0.762631	−0.237989	0.271407	0.105*	0.432 (16)
H72F	0.712747	−0.188141	0.344693	0.105*	0.432 (16)
C73	0.46293 (18)	0.11139 (16)	0.32544 (11)	0.0371 (5)	
N9	0.54009 (16)	0.09251 (14)	0.35388 (11)	0.0449 (5)	
C74	0.5761 (2)	0.15374 (17)	0.38172 (14)	0.0478 (6)	
C75	0.6692 (2)	0.1691 (2)	0.34225 (17)	0.0608 (8)	
C76	0.7139 (3)	0.2188 (2)	0.3733 (2)	0.0768 (10)	
H76	0.775449	0.230044	0.347570	0.092*	
C77	0.6687 (3)	0.2511 (2)	0.4410 (2)	0.0815 (11)	
H77	0.699474	0.284169	0.460721	0.098*	
C78	0.5783 (3)	0.2347 (2)	0.47949 (18)	0.0690 (9)	
H78	0.547801	0.257474	0.525202	0.083*	
C79	0.5313 (2)	0.18475 (18)	0.45160 (15)	0.0520 (7)	
C80	0.7237 (3)	0.1294 (3)	0.26970 (19)	0.0857 (11)	
H80A	0.789078	0.142530	0.252515	0.129*	
H80B	0.679167	0.155711	0.238954	0.129*	
H80C	0.737762	0.064399	0.271225	0.129*	
C81	0.4401 (2)	0.1584 (2)	0.49680 (15)	0.0638 (8)	
H81A	0.387461	0.167967	0.471652	0.096*	
H81B	0.410226	0.195206	0.538860	0.096*	
H81C	0.463872	0.095154	0.508977	0.096*	
O1	0.9343 (13)	0.1372 (13)	0.0105 (9)	0.228 (8)	0.552 (19)
C82	0.8985 (19)	0.1455 (18)	0.0847 (9)	0.180 (7)	0.552 (19)
H82A	0.919797	0.086408	0.106537	0.216*	0.552 (19)
H82B	0.822160	0.171249	0.099722	0.216*	0.552 (19)
C83	0.9497 (16)	0.2080 (14)	0.1033 (8)	0.154 (6)	0.552 (19)
H83A	0.994015	0.177455	0.134021	0.184*	0.552 (19)
H83B	0.896455	0.262114	0.127383	0.184*	0.552 (19)
C84	1.0165 (11)	0.2343 (12)	0.0345 (11)	0.181 (6)	0.552 (19)
H84A	0.981219	0.295282	0.020252	0.217*	0.552 (19)
H84B	1.085147	0.231743	0.039001	0.217*	0.552 (19)
C85	1.0264 (12)	0.1656 (19)	−0.0155 (9)	0.182 (9)	0.552 (19)
H85A	1.029009	0.191379	−0.062034	0.219*	0.552 (19)
H85B	1.090121	0.114196	−0.017944	0.219*	0.552 (19)
O1'	0.996 (3)	0.2346 (19)	0.0573 (12)	0.357 (16)	0.448 (19)
C82'	0.9945 (14)	0.2279 (10)	−0.0142 (9)	0.121 (5)	0.448 (19)
H82C	1.059140	0.231871	−0.047128	0.145*	0.448 (19)
H82D	0.934419	0.274420	−0.024916	0.145*	0.448 (19)
C83'	0.985 (3)	0.1349 (14)	−0.0142 (11)	0.202 (15)	0.448 (19)
H83C	1.053561	0.088211	−0.020926	0.242*	0.448 (19)
H83D	0.953558	0.128606	−0.050832	0.242*	0.448 (19)
C84'	0.914 (2)	0.1284 (19)	0.0577 (12)	0.178 (10)	0.448 (19)
H84C	0.841062	0.146918	0.056007	0.214*	0.448 (19)
H84D	0.933465	0.066102	0.074487	0.214*	0.448 (19)
C85'	0.928 (3)	0.190 (2)	0.1051 (11)	0.229 (16)	0.448 (19)
H85C	0.861217	0.234413	0.128114	0.275*	0.448 (19)
H85D	0.961376	0.156266	0.140532	0.275*	0.448 (19)
O2	0.0069 (11)	0.6883 (9)	0.2445 (8)	0.281 (7)	0.692 (11)
C86	0.0053 (11)	0.7656 (11)	0.2816 (7)	0.213 (6)	0.692 (11)
H86A	0.065590	0.750775	0.301101	0.256*	0.692 (11)
H86B	−0.059219	0.786982	0.318953	0.256*	0.692 (11)
C87	0.011 (2)	0.8350 (10)	0.2237 (11)	0.317 (14)	0.692 (11)
H87A	0.015446	0.891272	0.238782	0.380*	0.692 (11)
H87B	−0.045608	0.847792	0.200871	0.380*	0.692 (11)
C88	0.1153 (16)	0.7737 (13)	0.1806 (13)	0.293 (12)	0.692 (11)
H88A	0.169603	0.756091	0.205728	0.351*	0.692 (11)
H88B	0.140321	0.799965	0.135616	0.351*	0.692 (11)
C89	0.0748 (13)	0.6963 (11)	0.1735 (8)	0.232 (6)	0.692 (11)
H89A	0.034344	0.710457	0.138899	0.279*	0.692 (11)
H89B	0.132786	0.640049	0.159755	0.279*	0.692 (11)
O2'	0.0890 (18)	0.7946 (14)	0.2499 (12)	0.198 (9)	0.308 (11)
C86'	0.1048 (19)	0.733 (2)	0.1917 (18)	0.239 (19)	0.308 (11)
H86C	0.139241	0.670218	0.202345	0.287*	0.308 (11)
H86D	0.147548	0.749869	0.148331	0.287*	0.308 (11)
C87'	−0.007 (2)	0.7461 (15)	0.1857 (16)	0.188 (11)	0.308 (11)
H87C	−0.008325	0.720700	0.142863	0.225*	0.308 (11)
H87D	−0.051167	0.725750	0.226628	0.225*	0.308 (11)
C88'	−0.029 (2)	0.8471 (15)	0.1836 (14)	0.201 (11)	0.308 (11)
H88C	−0.097787	0.881231	0.177046	0.241*	0.308 (11)
H88D	0.026203	0.866060	0.150442	0.241*	0.308 (11)
C89'	−0.0235 (19)	0.846 (2)	0.2592 (13)	0.210 (15)	0.308 (11)
H89C	−0.041572	0.906993	0.277449	0.252*	0.308 (11)
H89D	−0.068652	0.814257	0.289556	0.252*	0.308 (11)

### {5,6-Bis(2,6-dimethylanilino)-3-(2,6-dimethylphenyl)-1,2,7-tris[(2,6-dimethylphenyl)imino]-3-azoniahept-3-ene-1,4,7-triido}tris(2,6-dimethylphenyl isocyanide)iron tetrahydrofuran disolvate (2) Atomic displacement parameters (Å^2^)

**Table:**

	*U*^11^	*U*^22^	*U*^33^	*U*^12^	*U*^13^	*U*^23^
Fe1	0.0388 (2)	0.0358 (2)	0.03504 (19)	−0.01454 (15)	−0.01060 (14)	0.00090 (14)
C1	0.0386 (13)	0.0378 (14)	0.0373 (14)	−0.0095 (11)	−0.0151 (11)	−0.0015 (11)
N1	0.045 (5)	0.051 (5)	0.027 (4)	−0.020 (3)	−0.015 (4)	−0.006 (4)
C2	0.050 (4)	0.044 (5)	0.038 (6)	−0.030 (3)	−0.008 (4)	−0.010 (5)
C3	0.069 (4)	0.049 (6)	0.042 (6)	−0.029 (4)	−0.023 (4)	0.003 (5)
C4	0.104 (6)	0.091 (8)	0.065 (8)	−0.049 (6)	−0.020 (6)	0.029 (6)
C5	0.099 (6)	0.126 (11)	0.100 (10)	−0.054 (7)	0.013 (7)	0.034 (8)
C6	0.065 (5)	0.091 (8)	0.101 (9)	−0.040 (5)	0.006 (5)	0.012 (7)
C7	0.055 (4)	0.056 (6)	0.057 (6)	−0.034 (4)	−0.011 (4)	−0.001 (5)
C8	0.071 (6)	0.078 (10)	0.059 (10)	−0.024 (6)	−0.027 (5)	0.019 (6)
C9	0.059 (8)	0.104 (10)	0.100 (10)	−0.042 (7)	−0.003 (6)	0.026 (8)
N1'	0.040 (3)	0.053 (4)	0.035 (5)	−0.028 (3)	−0.012 (4)	−0.002 (5)
C2'	0.051 (4)	0.046 (5)	0.030 (5)	−0.027 (3)	−0.008 (3)	−0.006 (4)
C3'	0.067 (4)	0.054 (5)	0.041 (6)	−0.034 (3)	−0.013 (4)	−0.004 (4)
C4'	0.097 (5)	0.080 (7)	0.064 (7)	−0.046 (5)	−0.020 (5)	0.028 (5)
C5'	0.081 (5)	0.121 (10)	0.095 (9)	−0.061 (6)	−0.002 (6)	0.045 (7)
C6'	0.057 (4)	0.098 (9)	0.088 (8)	−0.038 (5)	−0.004 (4)	0.018 (6)
C7'	0.047 (4)	0.065 (6)	0.055 (5)	−0.031 (4)	−0.011 (3)	−0.007 (4)
C8'	0.094 (7)	0.067 (7)	0.074 (12)	−0.027 (5)	−0.050 (6)	0.027 (6)
C9'	0.047 (7)	0.093 (8)	0.101 (9)	−0.016 (6)	−0.032 (8)	0.035 (7)
C10	0.0408 (14)	0.0392 (14)	0.0499 (15)	−0.0104 (11)	−0.0168 (12)	0.0030 (12)
N2	0.0618 (14)	0.0461 (13)	0.0544 (14)	−0.0100 (11)	−0.0253 (12)	−0.0097 (11)
C11	0.0696 (19)	0.0483 (17)	0.0621 (19)	0.0020 (15)	−0.0377 (17)	−0.0142 (14)
C12	0.094 (2)	0.0451 (18)	0.098 (3)	−0.0025 (17)	−0.062 (2)	−0.0181 (17)
C13	0.114 (3)	0.074 (3)	0.145 (4)	0.000 (2)	−0.071 (3)	−0.041 (3)
C14	0.114 (4)	0.092 (3)	0.122 (4)	0.013 (3)	−0.065 (3)	−0.060 (3)
C15	0.087 (3)	0.110 (3)	0.077 (3)	0.027 (2)	−0.038 (2)	−0.034 (2)
C16	0.070 (2)	0.073 (2)	0.063 (2)	0.0087 (16)	−0.0324 (18)	−0.0187 (17)
C17	0.109 (3)	0.081 (3)	0.123 (3)	−0.043 (2)	−0.054 (3)	0.015 (2)
C18	0.070 (2)	0.113 (3)	0.063 (2)	−0.008 (2)	−0.0109 (17)	0.000 (2)
C19	0.0455 (15)	0.0394 (14)	0.0482 (15)	−0.0151 (12)	−0.0165 (12)	0.0030 (12)
N3	0.0548 (14)	0.0583 (15)	0.0595 (15)	−0.0268 (12)	−0.0084 (12)	0.0162 (12)
C20	0.0568 (18)	0.064 (2)	0.076 (2)	−0.0296 (16)	−0.0067 (16)	0.0218 (16)
C21	0.096 (3)	0.101 (3)	0.072 (2)	−0.045 (2)	−0.004 (2)	0.027 (2)
C22	0.175 (5)	0.154 (5)	0.089 (3)	−0.086 (4)	0.003 (3)	0.037 (3)
C23	0.167 (6)	0.176 (6)	0.146 (5)	−0.114 (5)	−0.006 (4)	0.045 (5)
C24	0.124 (4)	0.136 (4)	0.157 (5)	−0.095 (3)	−0.038 (4)	0.039 (4)
C25	0.093 (3)	0.092 (3)	0.116 (3)	−0.056 (2)	−0.031 (2)	0.026 (2)
C26	0.185 (5)	0.134 (4)	0.085 (3)	−0.081 (4)	−0.037 (3)	0.012 (3)
C27	0.176 (5)	0.120 (4)	0.148 (5)	−0.083 (4)	−0.069 (4)	0.005 (3)
C28	0.0436 (14)	0.0416 (15)	0.0379 (13)	−0.0155 (12)	−0.0112 (11)	0.0030 (11)
N4	0.0595 (14)	0.0379 (13)	0.0616 (14)	−0.0123 (11)	−0.0306 (12)	0.0032 (10)
C29	0.0577 (17)	0.0364 (15)	0.074 (2)	−0.0072 (13)	−0.0371 (15)	0.0009 (14)
C30	0.071 (2)	0.0430 (17)	0.089 (2)	−0.0169 (15)	−0.0421 (18)	0.0078 (15)
C31	0.094 (3)	0.052 (2)	0.120 (3)	−0.0301 (19)	−0.052 (2)	0.005 (2)
C32	0.107 (3)	0.056 (2)	0.128 (4)	−0.022 (2)	−0.058 (3)	−0.017 (2)
C33	0.084 (2)	0.059 (2)	0.088 (2)	−0.0017 (18)	−0.039 (2)	−0.0197 (18)
C34	0.0621 (18)	0.0437 (16)	0.077 (2)	−0.0026 (14)	−0.0342 (17)	−0.0060 (15)
C35	0.102 (3)	0.069 (2)	0.097 (3)	−0.035 (2)	−0.029 (2)	0.014 (2)
C36	0.069 (2)	0.070 (2)	0.083 (2)	−0.0125 (18)	−0.0158 (18)	−0.0082 (18)
C37	0.0437 (14)	0.0391 (14)	0.0364 (13)	−0.0140 (11)	−0.0115 (11)	0.0033 (11)
N5	0.0586 (14)	0.0451 (13)	0.0492 (13)	−0.0113 (11)	−0.0275 (11)	0.0027 (10)
C38	0.0644 (19)	0.0472 (17)	0.0592 (18)	−0.0090 (14)	−0.0372 (15)	0.0014 (14)
C39	0.065 (2)	0.061 (2)	0.083 (2)	−0.0084 (17)	−0.0372 (18)	−0.0034 (17)
C40	0.077 (3)	0.077 (3)	0.133 (4)	0.002 (2)	−0.049 (3)	−0.001 (3)
C41	0.122 (4)	0.069 (3)	0.168 (5)	0.010 (3)	−0.084 (4)	0.023 (3)
C42	0.140 (4)	0.066 (3)	0.119 (3)	−0.019 (3)	−0.073 (3)	0.033 (2)
C43	0.094 (2)	0.0542 (19)	0.066 (2)	−0.0192 (18)	−0.0384 (19)	0.0095 (16)
C44	0.078 (2)	0.096 (3)	0.094 (3)	−0.018 (2)	−0.012 (2)	−0.002 (2)
C45	0.133 (4)	0.079 (3)	0.074 (2)	−0.038 (2)	−0.016 (2)	0.013 (2)
C46	0.0359 (12)	0.0412 (14)	0.0308 (12)	−0.0159 (11)	−0.0067 (10)	0.0050 (10)
N6	0.0468 (12)	0.0354 (11)	0.0381 (11)	−0.0141 (9)	−0.0172 (9)	0.0030 (9)
C47	0.0604 (17)	0.0382 (14)	0.0520 (16)	−0.0130 (13)	−0.0311 (13)	0.0006 (12)
C48	0.070 (2)	0.0504 (17)	0.080 (2)	−0.0257 (15)	−0.0369 (17)	0.0079 (15)
C49	0.110 (3)	0.075 (3)	0.130 (4)	−0.050 (2)	−0.064 (3)	0.009 (2)
C50	0.157 (4)	0.078 (3)	0.131 (4)	−0.047 (3)	−0.092 (4)	−0.004 (3)
C51	0.135 (3)	0.068 (2)	0.071 (2)	−0.013 (2)	−0.062 (2)	−0.0150 (18)
C52	0.081 (2)	0.0481 (17)	0.0518 (17)	−0.0080 (15)	−0.0346 (16)	−0.0027 (13)
C53	0.072 (2)	0.076 (2)	0.095 (3)	−0.0385 (18)	−0.0188 (19)	0.0206 (19)
C54	0.099 (3)	0.076 (2)	0.0475 (18)	−0.011 (2)	−0.0131 (17)	−0.0015 (16)
C55	0.0414 (13)	0.0350 (13)	0.0382 (13)	−0.0137 (11)	−0.0110 (11)	−0.0014 (10)
N7	0.0504 (14)	0.0394 (12)	0.0521 (13)	−0.0082 (11)	−0.0205 (11)	−0.0082 (10)
C56	0.0673 (19)	0.0462 (16)	0.0334 (14)	−0.0023 (14)	−0.0184 (13)	−0.0028 (12)
C57	0.101 (2)	0.0456 (17)	0.0453 (16)	−0.0033 (17)	−0.0291 (17)	−0.0051 (13)
C58	0.132 (4)	0.056 (2)	0.062 (2)	0.012 (2)	−0.034 (2)	−0.0182 (17)
C59	0.107 (3)	0.096 (3)	0.070 (3)	0.038 (3)	−0.032 (2)	−0.023 (2)
C60	0.072 (2)	0.120 (3)	0.053 (2)	0.008 (2)	−0.0155 (17)	−0.014 (2)
C61	0.0622 (19)	0.076 (2)	0.0361 (15)	−0.0035 (17)	−0.0137 (14)	−0.0047 (14)
C62	0.138 (3)	0.0514 (19)	0.071 (2)	−0.033 (2)	−0.039 (2)	−0.0058 (16)
C63	0.063 (2)	0.109 (3)	0.0546 (19)	−0.0346 (19)	−0.0045 (15)	−0.0038 (18)
C64	0.0344 (12)	0.0390 (14)	0.0344 (13)	−0.0117 (11)	−0.0065 (10)	0.0018 (10)
N8	0.059 (4)	0.029 (3)	0.035 (5)	−0.013 (3)	−0.015 (3)	0.001 (3)
C65	0.063 (4)	0.037 (4)	0.035 (4)	−0.021 (3)	−0.017 (5)	0.005 (3)
C66	0.073 (6)	0.046 (4)	0.040 (4)	−0.018 (4)	−0.012 (6)	0.000 (4)
C67	0.092 (10)	0.046 (6)	0.079 (4)	−0.029 (6)	−0.039 (8)	0.014 (6)
C68	0.104 (10)	0.039 (4)	0.087 (4)	−0.020 (5)	−0.035 (7)	0.008 (3)
C69	0.098 (7)	0.044 (4)	0.076 (4)	−0.007 (5)	−0.033 (6)	0.003 (3)
C70	0.075 (6)	0.040 (4)	0.050 (3)	−0.009 (4)	−0.030 (6)	0.001 (4)
C71	0.070 (7)	0.068 (9)	0.070 (5)	−0.037 (6)	−0.021 (5)	0.005 (6)
C72	0.051 (5)	0.084 (11)	0.094 (7)	−0.001 (5)	−0.024 (4)	0.004 (6)
N8'	0.037 (4)	0.054 (6)	0.039 (7)	−0.011 (4)	−0.019 (4)	−0.004 (4)
C65'	0.073 (7)	0.034 (5)	0.043 (5)	−0.015 (4)	−0.016 (7)	0.004 (5)
C66'	0.071 (7)	0.047 (6)	0.048 (6)	−0.034 (6)	−0.017 (8)	0.006 (6)
C67'	0.104 (10)	0.058 (7)	0.075 (5)	−0.032 (7)	−0.042 (10)	0.013 (6)
C68'	0.108 (11)	0.045 (6)	0.090 (6)	−0.025 (7)	−0.041 (8)	0.007 (5)
C69'	0.093 (10)	0.043 (6)	0.084 (5)	−0.014 (6)	−0.033 (8)	0.003 (5)
C70'	0.078 (6)	0.042 (5)	0.055 (4)	−0.021 (4)	−0.034 (5)	−0.002 (5)
C71'	0.063 (7)	0.065 (11)	0.076 (7)	−0.029 (6)	−0.002 (6)	0.008 (8)
C72'	0.071 (9)	0.066 (10)	0.065 (6)	0.002 (7)	−0.034 (7)	0.001 (6)
C73	0.0369 (13)	0.0428 (14)	0.0319 (12)	−0.0157 (11)	−0.0068 (10)	0.0034 (10)
N9	0.0461 (12)	0.0450 (12)	0.0494 (12)	−0.0170 (10)	−0.0198 (10)	0.0033 (10)
C74	0.0513 (16)	0.0419 (15)	0.0604 (17)	−0.0159 (12)	−0.0324 (13)	0.0101 (12)
C75	0.0538 (17)	0.0613 (19)	0.077 (2)	−0.0240 (15)	−0.0286 (16)	0.0111 (16)
C76	0.065 (2)	0.071 (2)	0.114 (3)	−0.0345 (18)	−0.043 (2)	0.017 (2)
C77	0.093 (3)	0.057 (2)	0.125 (3)	−0.0309 (19)	−0.071 (3)	0.007 (2)
C78	0.086 (2)	0.0534 (19)	0.082 (2)	−0.0179 (17)	−0.0511 (19)	−0.0015 (16)
C79	0.0629 (17)	0.0422 (15)	0.0589 (17)	−0.0137 (13)	−0.0345 (14)	0.0063 (13)
C80	0.061 (2)	0.107 (3)	0.093 (3)	−0.038 (2)	−0.0128 (19)	0.005 (2)
C81	0.074 (2)	0.065 (2)	0.0521 (17)	−0.0163 (16)	−0.0250 (15)	0.0024 (14)
O1	0.217 (14)	0.334 (16)	0.126 (12)	−0.104 (11)	0.013 (12)	−0.093 (12)
C82	0.171 (14)	0.232 (19)	0.118 (13)	−0.045 (13)	−0.007 (11)	−0.078 (12)
C83	0.163 (12)	0.159 (14)	0.130 (9)	0.007 (10)	−0.092 (9)	−0.008 (9)
C84	0.109 (9)	0.251 (17)	0.169 (13)	−0.009 (9)	−0.076 (9)	0.008 (10)
C85	0.112 (9)	0.28 (3)	0.113 (10)	−0.007 (14)	−0.023 (8)	−0.016 (14)
O1'	0.59 (4)	0.44 (3)	0.205 (18)	−0.32 (3)	−0.17 (2)	0.000 (17)
C82'	0.113 (11)	0.137 (11)	0.129 (10)	−0.035 (9)	−0.063 (9)	0.004 (9)
C83'	0.26 (4)	0.199 (17)	0.121 (14)	−0.11 (2)	0.047 (18)	−0.035 (13)
C84'	0.23 (2)	0.175 (18)	0.087 (16)	−0.052 (15)	0.024 (17)	−0.031 (15)
C85'	0.31 (4)	0.20 (3)	0.123 (14)	−0.01 (2)	−0.050 (18)	−0.050 (14)
O2	0.221 (10)	0.248 (12)	0.376 (17)	−0.092 (10)	−0.084 (10)	0.083 (11)
C86	0.142 (9)	0.235 (15)	0.243 (13)	−0.042 (11)	−0.066 (9)	0.092 (10)
C87	0.44 (4)	0.198 (16)	0.30 (3)	−0.09 (2)	−0.13 (2)	0.120 (13)
C88	0.35 (3)	0.33 (2)	0.27 (2)	−0.25 (2)	−0.048 (16)	0.109 (17)
C89	0.236 (18)	0.226 (14)	0.284 (14)	−0.091 (12)	−0.142 (12)	0.060 (12)
O2'	0.235 (19)	0.166 (17)	0.200 (19)	−0.070 (14)	−0.082 (17)	0.070 (13)
C86'	0.23 (2)	0.22 (3)	0.29 (4)	−0.02 (2)	−0.17 (3)	0.02 (2)
C87'	0.17 (2)	0.153 (16)	0.21 (3)	0.026 (15)	−0.092 (19)	0.015 (16)
C88'	0.25 (3)	0.178 (16)	0.184 (19)	−0.053 (17)	−0.11 (2)	0.057 (19)
C89'	0.23 (2)	0.26 (4)	0.16 (2)	−0.09 (2)	−0.084 (19)	0.02 (2)

### {5,6-Bis(2,6-dimethylanilino)-3-(2,6-dimethylphenyl)-1,2,7-tris[(2,6-dimethylphenyl)imino]-3-azoniahept-3-ene-1,4,7-triido}tris(2,6-dimethylphenyl isocyanide)iron tetrahydrofuran disolvate (2) Geometric parameters (Å, º)

**Table:**

Fe1—C10	1.851 (3)	C53—H53C	0.9600
Fe1—C1	1.852 (2)	C54—H54A	0.9600
Fe1—C19	1.854 (3)	C54—H54B	0.9600
Fe1—C46	1.955 (2)	C54—H54C	0.9600
Fe1—C73	2.011 (2)	C55—C64	1.390 (3)
Fe1—C28	2.014 (2)	C55—N7	1.418 (3)
C1—N1'	1.177 (6)	N7—C56	1.403 (3)
C1—N1	1.178 (7)	N7—H7	0.85 (3)
N1—C2	1.407 (10)	C56—C61	1.405 (4)
C2—C3	1.388 (10)	C56—C57	1.408 (4)
C2—C7	1.401 (11)	C57—C58	1.388 (5)
C3—C4	1.381 (11)	C57—C62	1.500 (5)
C3—C8	1.506 (8)	C58—C59	1.367 (6)
C4—C5	1.365 (12)	C58—H58	0.9300
C4—H4	0.9300	C59—C60	1.370 (6)
C5—C6	1.363 (12)	C59—H59	0.9300
C5—H5	0.9300	C60—C61	1.393 (4)
C6—C7	1.376 (11)	C60—H60	0.9300
C6—H6	0.9300	C61—C63	1.497 (4)
C7—C9	1.513 (8)	C62—H62A	0.9600
C8—H8A	0.9600	C62—H62B	0.9600
C8—H8B	0.9600	C62—H62C	0.9600
C8—H8C	0.9600	C63—H63A	0.9600
C9—H9A	0.9600	C63—H63B	0.9600
C9—H9B	0.9600	C63—H63C	0.9600
C9—H9C	0.9600	C64—N8'	1.345 (7)
N1'—C2'	1.406 (9)	C64—N8	1.348 (6)
C2'—C3'	1.387 (10)	C64—C73	1.523 (3)
C2'—C7'	1.401 (10)	N8—C65	1.438 (10)
C3'—C4'	1.382 (10)	N8—H8	0.8600
C3'—C8'	1.508 (8)	C65—C66	1.387 (10)
C4'—C5'	1.368 (11)	C65—C70	1.408 (10)
C4'—H4'	0.9300	C66—C67	1.405 (10)
C5'—C6'	1.364 (10)	C66—C71	1.497 (7)
C5'—H5'	0.9300	C67—C68	1.373 (11)
C6'—C7'	1.364 (10)	C67—H67	0.9300
C6'—H6'	0.9300	C68—C69	1.387 (11)
C7'—C9'	1.511 (8)	C68—H68	0.9300
C8'—H8D	0.9600	C69—C70	1.394 (10)
C8'—H8E	0.9600	C69—H69	0.9300
C8'—H8F	0.9600	C70—C72	1.498 (7)
C9'—H9D	0.9600	C71—H71A	0.9600
C9'—H9E	0.9600	C71—H71B	0.9600
C9'—H9F	0.9600	C71—H71C	0.9600
C10—N2	1.172 (3)	C72—H72A	0.9600
N2—C11	1.386 (3)	C72—H72B	0.9600
C11—C16	1.369 (4)	C72—H72C	0.9600
C11—C12	1.429 (4)	N8'—C65'	1.428 (12)
C12—C13	1.369 (5)	N8'—H8'	0.8600
C12—C17	1.483 (5)	C65'—C66'	1.384 (12)
C13—C14	1.338 (6)	C65'—C70'	1.407 (12)
C13—H13	0.9300	C66'—C67'	1.396 (12)
C14—C15	1.393 (6)	C66'—C71'	1.493 (8)
C14—H14	0.9300	C67'—C68'	1.367 (13)
C15—C16	1.424 (5)	C67'—H67'	0.9300
C15—H15	0.9300	C68'—C69'	1.394 (14)
C16—C18	1.493 (5)	C68'—H68'	0.9300
C17—H17A	0.9600	C69'—C70'	1.399 (13)
C17—H17B	0.9600	C69'—H69'	0.9300
C17—H17C	0.9600	C70'—C72'	1.499 (8)
C18—H18A	0.9600	C71'—H71D	0.9600
C18—H18B	0.9600	C71'—H71E	0.9600
C18—H18C	0.9600	C71'—H71F	0.9600
C19—N3	1.170 (3)	C72'—H72D	0.9600
N3—C20	1.411 (3)	C72'—H72E	0.9600
C20—C25	1.374 (5)	C72'—H72F	0.9600
C20—C21	1.404 (5)	C73—N9	1.285 (3)
C21—C22	1.378 (5)	N9—C74	1.415 (3)
C21—C26	1.487 (5)	C74—C75	1.398 (4)
C22—C23	1.339 (7)	C74—C79	1.403 (4)
C22—H22	0.9300	C75—C76	1.398 (4)
C23—C24	1.351 (7)	C75—C80	1.501 (4)
C23—H23	0.9300	C76—C77	1.371 (5)
C24—C25	1.426 (6)	C76—H76	0.9300
C24—H24	0.9300	C77—C78	1.368 (5)
C25—C27	1.483 (6)	C77—H77	0.9300
C26—H26A	0.9600	C78—C79	1.390 (4)
C26—H26B	0.9600	C78—H78	0.9300
C26—H26C	0.9600	C79—C81	1.492 (4)
C27—H27A	0.9600	C80—H80A	0.9600
C27—H27B	0.9600	C80—H80B	0.9600
C27—H27C	0.9600	C80—H80C	0.9600
C28—N4	1.288 (3)	C81—H81A	0.9600
C28—C37	1.530 (3)	C81—H81B	0.9600
N4—C29	1.418 (3)	C81—H81C	0.9600
C29—C34	1.396 (4)	O1—C82	1.416 (11)
C29—C30	1.397 (4)	O1—C85	1.436 (12)
C30—C31	1.388 (4)	C82—C83	1.495 (11)
C30—C35	1.504 (5)	C82—H82A	0.9700
C31—C32	1.373 (5)	C82—H82B	0.9700
C31—H31	0.9300	C83—C84	1.530 (13)
C32—C33	1.372 (5)	C83—H83A	0.9700
C32—H32	0.9300	C83—H83B	0.9700
C33—C34	1.397 (4)	C84—C85	1.461 (12)
C33—H33	0.9300	C84—H84A	0.9700
C34—C36	1.494 (4)	C84—H84B	0.9700
C35—H35A	0.9600	C85—H85A	0.9700
C35—H35B	0.9600	C85—H85B	0.9700
C35—H35C	0.9600	O1'—C82'	1.436 (12)
C36—H36A	0.9600	O1'—C85'	1.444 (13)
C36—H36B	0.9600	C82'—C83'	1.489 (13)
C36—H36C	0.9600	C82'—H82C	0.9700
C37—N5	1.261 (3)	C82'—H82D	0.9700
C37—N6	1.429 (3)	C83'—C84'	1.508 (14)
N5—C38	1.412 (3)	C83'—H83C	0.9700
C38—C39	1.399 (4)	C83'—H83D	0.9700
C38—C43	1.399 (4)	C84'—C85'	1.487 (12)
C39—C40	1.384 (5)	C84'—H84C	0.9700
C39—C44	1.498 (5)	C84'—H84D	0.9700
C40—C41	1.359 (6)	C85'—H85C	0.9700
C40—H40	0.9300	C85'—H85D	0.9700
C41—C42	1.378 (6)	O2—C86	1.445 (11)
C41—H41	0.9300	O2—C89	1.482 (11)
C42—C43	1.385 (5)	C86—C87	1.510 (12)
C42—H42	0.9300	C86—H86A	0.9700
C43—C45	1.497 (5)	C86—H86B	0.9700
C44—H44A	0.9600	C87—C88	1.510 (15)
C44—H44B	0.9600	C87—H87A	0.9700
C44—H44C	0.9600	C87—H87B	0.9700
C45—H45A	0.9600	C88—C89	1.511 (12)
C45—H45B	0.9600	C88—H88A	0.9700
C45—H45C	0.9600	C88—H88B	0.9700
C46—N6	1.354 (3)	C89—H89A	0.9700
C46—C55	1.414 (3)	C89—H89B	0.9700
N6—C47	1.459 (3)	O2'—C89'	1.469 (13)
C47—C48	1.389 (4)	O2'—C86'	1.477 (13)
C47—C52	1.398 (4)	C86'—C87'	1.539 (13)
C48—C49	1.388 (4)	C86'—H86C	0.9700
C48—C53	1.497 (4)	C86'—H86D	0.9700
C49—C50	1.366 (6)	C87'—C88'	1.492 (15)
C49—H49	0.9300	C87'—H87C	0.9700
C50—C51	1.365 (6)	C87'—H87D	0.9700
C50—H50	0.9300	C88'—C89'	1.515 (14)
C51—C52	1.395 (4)	C88'—H88C	0.9700
C51—H51	0.9300	C88'—H88D	0.9700
C52—C54	1.486 (4)	C89'—H89C	0.9700
C53—H53A	0.9600	C89'—H89D	0.9700
C53—H53B	0.9600		
			
C10—Fe1—C1	84.67 (10)	C52—C54—H54C	109.5
C10—Fe1—C19	94.05 (11)	H54A—C54—H54C	109.5
C1—Fe1—C19	177.81 (11)	H54B—C54—H54C	109.5
C10—Fe1—C46	171.45 (10)	C64—C55—C46	111.4 (2)
C1—Fe1—C46	86.80 (9)	C64—C55—N7	122.4 (2)
C19—Fe1—C46	94.46 (10)	C46—C55—N7	126.2 (2)
C10—Fe1—C73	97.51 (10)	C56—N7—C55	127.5 (2)
C1—Fe1—C73	88.02 (9)	C56—N7—H7	118.2 (17)
C19—Fe1—C73	90.39 (10)	C55—N7—H7	114.2 (17)
C46—Fe1—C73	81.56 (9)	N7—C56—C61	122.5 (3)
C10—Fe1—C28	99.78 (10)	N7—C56—C57	116.8 (3)
C1—Fe1—C28	96.36 (10)	C61—C56—C57	120.7 (3)
C19—Fe1—C28	85.61 (10)	C58—C57—C56	118.2 (4)
C46—Fe1—C28	81.76 (10)	C58—C57—C62	120.5 (3)
C73—Fe1—C28	162.48 (9)	C56—C57—C62	121.3 (3)
N1'—C1—Fe1	175.6 (11)	C59—C58—C57	121.5 (4)
N1—C1—Fe1	168.3 (11)	C59—C58—H58	119.3
C1—N1—C2	172 (2)	C57—C58—H58	119.3
C3—C2—C7	122.9 (10)	C58—C59—C60	119.8 (4)
C3—C2—N1	118.3 (13)	C58—C59—H59	120.1
C7—C2—N1	118.8 (13)	C60—C59—H59	120.1
C4—C3—C2	116.8 (11)	C59—C60—C61	121.8 (4)
C4—C3—C8	116.5 (17)	C59—C60—H60	119.1
C2—C3—C8	126.7 (18)	C61—C60—H60	119.1
C5—C4—C3	121.5 (12)	C60—C61—C56	117.6 (3)
C5—C4—H4	119.2	C60—C61—C63	118.1 (3)
C3—C4—H4	119.2	C56—C61—C63	124.2 (3)
C6—C5—C4	120.3 (12)	C57—C62—H62A	109.5
C6—C5—H5	119.8	C57—C62—H62B	109.5
C4—C5—H5	119.8	H62A—C62—H62B	109.5
C5—C6—C7	121.6 (12)	C57—C62—H62C	109.5
C5—C6—H6	119.2	H62A—C62—H62C	109.5
C7—C6—H6	119.2	H62B—C62—H62C	109.5
C6—C7—C2	116.8 (11)	C61—C63—H63A	109.5
C6—C7—C9	115.8 (17)	C61—C63—H63B	109.5
C2—C7—C9	127.2 (16)	H63A—C63—H63B	109.5
C3—C8—H8A	109.5	C61—C63—H63C	109.5
C3—C8—H8B	109.5	H63A—C63—H63C	109.5
H8A—C8—H8B	109.5	H63B—C63—H63C	109.5
C3—C8—H8C	109.5	N8'—C64—C55	125.1 (10)
H8A—C8—H8C	109.5	N8—C64—C55	132.9 (7)
H8B—C8—H8C	109.5	N8'—C64—C73	119.1 (9)
C7—C9—H9A	109.5	N8—C64—C73	111.8 (7)
C7—C9—H9B	109.5	C55—C64—C73	115.3 (2)
H9A—C9—H9B	109.5	C64—N8—C65	129.4 (19)
C7—C9—H9C	109.5	C64—N8—H8	115.3
H9A—C9—H9C	109.5	C65—N8—H8	115.3
H9B—C9—H9C	109.5	C66—C65—C70	121.4 (9)
C1—N1'—C2'	172.4 (18)	C66—C65—N8	122.7 (11)
C3'—C2'—C7'	122.8 (9)	C70—C65—N8	115.1 (10)
C3'—C2'—N1'	119.8 (12)	C65—C66—C67	119.8 (9)
C7'—C2'—N1'	117.4 (12)	C65—C66—C71	122.0 (11)
C4'—C3'—C2'	116.8 (11)	C67—C66—C71	118.0 (11)
C4'—C3'—C8'	123.2 (16)	C68—C67—C66	118.9 (9)
C2'—C3'—C8'	119.9 (16)	C68—C67—H67	120.5
C5'—C4'—C3'	121.5 (11)	C66—C67—H67	120.5
C5'—C4'—H4'	119.2	C67—C68—C69	121.2 (9)
C3'—C4'—H4'	119.2	C67—C68—H68	119.4
C6'—C5'—C4'	119.9 (10)	C69—C68—H68	119.4
C6'—C5'—H5'	120.1	C68—C69—C70	121.1 (9)
C4'—C5'—H5'	120.1	C68—C69—H69	119.5
C5'—C6'—C7'	122.0 (11)	C70—C69—H69	119.5
C5'—C6'—H6'	119.0	C69—C70—C65	117.2 (9)
C7'—C6'—H6'	119.0	C69—C70—C72	119.4 (11)
C6'—C7'—C2'	116.9 (10)	C65—C70—C72	123.3 (11)
C6'—C7'—C9'	125.3 (16)	C66—C71—H71A	109.5
C2'—C7'—C9'	117.6 (14)	C66—C71—H71B	109.5
C3'—C8'—H8D	109.5	H71A—C71—H71B	109.5
C3'—C8'—H8E	109.5	C66—C71—H71C	109.5
H8D—C8'—H8E	109.5	H71A—C71—H71C	109.5
C3'—C8'—H8F	109.5	H71B—C71—H71C	109.5
H8D—C8'—H8F	109.5	C70—C72—H72A	109.5
H8E—C8'—H8F	109.5	C70—C72—H72B	109.5
C7'—C9'—H9D	109.5	H72A—C72—H72B	109.5
C7'—C9'—H9E	109.5	C70—C72—H72C	109.5
H9D—C9'—H9E	109.5	H72A—C72—H72C	109.5
C7'—C9'—H9F	109.5	H72B—C72—H72C	109.5
H9D—C9'—H9F	109.5	C64—N8'—C65'	136 (2)
H9E—C9'—H9F	109.5	C64—N8'—H8'	112.1
N2—C10—Fe1	170.3 (2)	C65'—N8'—H8'	112.1
C10—N2—C11	177.8 (3)	C66'—C65'—C70'	124.6 (12)
C16—C11—N2	119.7 (3)	C66'—C65'—N8'	120.4 (13)
C16—C11—C12	124.2 (3)	C70'—C65'—N8'	115.0 (13)
N2—C11—C12	116.1 (3)	C65'—C66'—C67'	116.1 (12)
C13—C12—C11	116.0 (4)	C65'—C66'—C71'	126.0 (14)
C13—C12—C17	122.7 (4)	C67'—C66'—C71'	117.9 (14)
C11—C12—C17	121.2 (3)	C68'—C67'—C66'	120.5 (14)
C14—C13—C12	121.9 (4)	C68'—C67'—H67'	119.7
C14—C13—H13	119.0	C66'—C67'—H67'	119.7
C12—C13—H13	119.0	C67'—C68'—C69'	123.1 (13)
C13—C14—C15	122.4 (4)	C67'—C68'—H68'	118.5
C13—C14—H14	118.8	C69'—C68'—H68'	118.5
C15—C14—H14	118.8	C68'—C69'—C70'	118.0 (11)
C14—C15—C16	118.9 (4)	C68'—C69'—H69'	121.0
C14—C15—H15	120.5	C70'—C69'—H69'	121.0
C16—C15—H15	120.5	C69'—C70'—C65'	117.4 (13)
C11—C16—C15	116.5 (4)	C69'—C70'—C72'	122.6 (15)
C11—C16—C18	122.7 (3)	C65'—C70'—C72'	119.7 (16)
C15—C16—C18	120.8 (4)	C66'—C71'—H71D	109.5
C12—C17—H17A	109.5	C66'—C71'—H71E	109.5
C12—C17—H17B	109.5	H71D—C71'—H71E	109.5
H17A—C17—H17B	109.5	C66'—C71'—H71F	109.5
C12—C17—H17C	109.5	H71D—C71'—H71F	109.5
H17A—C17—H17C	109.5	H71E—C71'—H71F	109.5
H17B—C17—H17C	109.5	C70'—C72'—H72D	109.5
C16—C18—H18A	109.5	C70'—C72'—H72E	109.5
C16—C18—H18B	109.5	H72D—C72'—H72E	109.5
H18A—C18—H18B	109.5	C70'—C72'—H72F	109.5
C16—C18—H18C	109.5	H72D—C72'—H72F	109.5
H18A—C18—H18C	109.5	H72E—C72'—H72F	109.5
H18B—C18—H18C	109.5	N9—C73—C64	110.2 (2)
N3—C19—Fe1	178.3 (2)	N9—C73—Fe1	140.42 (18)
C19—N3—C20	174.2 (3)	C64—C73—Fe1	109.28 (15)
C25—C20—C21	122.9 (3)	C73—N9—C74	128.2 (2)
C25—C20—N3	119.1 (3)	C75—C74—C79	120.4 (2)
C21—C20—N3	118.0 (3)	C75—C74—N9	117.8 (2)
C22—C21—C20	117.1 (4)	C79—C74—N9	120.7 (2)
C22—C21—C26	121.3 (4)	C74—C75—C76	118.4 (3)
C20—C21—C26	121.6 (3)	C74—C75—C80	121.3 (3)
C23—C22—C21	120.9 (5)	C76—C75—C80	120.3 (3)
C23—C22—H22	119.6	C77—C76—C75	121.3 (3)
C21—C22—H22	119.6	C77—C76—H76	119.4
C22—C23—C24	123.1 (5)	C75—C76—H76	119.4
C22—C23—H23	118.5	C78—C77—C76	119.9 (3)
C24—C23—H23	118.5	C78—C77—H77	120.0
C23—C24—C25	119.1 (5)	C76—C77—H77	120.0
C23—C24—H24	120.4	C77—C78—C79	121.3 (3)
C25—C24—H24	120.4	C77—C78—H78	119.4
C20—C25—C24	117.0 (4)	C79—C78—H78	119.4
C20—C25—C27	121.1 (3)	C78—C79—C74	118.7 (3)
C24—C25—C27	122.0 (4)	C78—C79—C81	120.5 (3)
C21—C26—H26A	109.5	C74—C79—C81	120.7 (2)
C21—C26—H26B	109.5	C75—C80—H80A	109.5
H26A—C26—H26B	109.5	C75—C80—H80B	109.5
C21—C26—H26C	109.5	H80A—C80—H80B	109.5
H26A—C26—H26C	109.5	C75—C80—H80C	109.5
H26B—C26—H26C	109.5	H80A—C80—H80C	109.5
C25—C27—H27A	109.5	H80B—C80—H80C	109.5
C25—C27—H27B	109.5	C79—C81—H81A	109.5
H27A—C27—H27B	109.5	C79—C81—H81B	109.5
C25—C27—H27C	109.5	H81A—C81—H81B	109.5
H27A—C27—H27C	109.5	C79—C81—H81C	109.5
H27B—C27—H27C	109.5	H81A—C81—H81C	109.5
N4—C28—C37	109.9 (2)	H81B—C81—H81C	109.5
N4—C28—Fe1	137.80 (19)	C82—O1—C85	110.7 (14)
C37—C28—Fe1	111.97 (16)	O1—C82—C83	104.8 (12)
C28—N4—C29	125.0 (2)	O1—C82—H82A	110.8
C34—C29—C30	120.6 (3)	C83—C82—H82A	110.8
C34—C29—N4	119.9 (3)	O1—C82—H82B	110.8
C30—C29—N4	118.8 (3)	C83—C82—H82B	110.8
C31—C30—C29	118.6 (3)	H82A—C82—H82B	108.9
C31—C30—C35	120.2 (3)	C82—C83—C84	107.1 (10)
C29—C30—C35	121.1 (3)	C82—C83—H83A	110.3
C32—C31—C30	121.3 (3)	C84—C83—H83A	110.3
C32—C31—H31	119.3	C82—C83—H83B	110.3
C30—C31—H31	119.3	C84—C83—H83B	110.3
C33—C32—C31	119.8 (3)	H83A—C83—H83B	108.5
C33—C32—H32	120.1	C85—C84—C83	103.3 (11)
C31—C32—H32	120.1	C85—C84—H84A	111.1
C32—C33—C34	121.0 (3)	C83—C84—H84A	111.1
C32—C33—H33	119.5	C85—C84—H84B	111.1
C34—C33—H33	119.5	C83—C84—H84B	111.1
C29—C34—C33	118.6 (3)	H84A—C84—H84B	109.1
C29—C34—C36	120.7 (3)	O1—C85—C84	105.8 (11)
C33—C34—C36	120.7 (3)	O1—C85—H85A	110.6
C30—C35—H35A	109.5	C84—C85—H85A	110.6
C30—C35—H35B	109.5	O1—C85—H85B	110.6
H35A—C35—H35B	109.5	C84—C85—H85B	110.6
C30—C35—H35C	109.5	H85A—C85—H85B	108.7
H35A—C35—H35C	109.5	C82'—O1'—C85'	111.9 (15)
H35B—C35—H35C	109.5	O1'—C82'—C83'	100.4 (14)
C34—C36—H36A	109.5	O1'—C82'—H82C	111.7
C34—C36—H36B	109.5	C83'—C82'—H82C	111.7
H36A—C36—H36B	109.5	O1'—C82'—H82D	111.7
C34—C36—H36C	109.5	C83'—C82'—H82D	111.7
H36A—C36—H36C	109.5	H82C—C82'—H82D	109.5
H36B—C36—H36C	109.5	C82'—C83'—C84'	103.5 (12)
N5—C37—N6	116.3 (2)	C82'—C83'—H83C	111.1
N5—C37—C28	132.5 (2)	C84'—C83'—H83C	111.1
N6—C37—C28	111.2 (2)	C82'—C83'—H83D	111.1
C37—N5—C38	127.4 (2)	C84'—C83'—H83D	111.1
C39—C38—C43	120.4 (3)	H83C—C83'—H83D	109.0
C39—C38—N5	119.8 (3)	C85'—C84'—C83'	107.0 (12)
C43—C38—N5	118.9 (3)	C85'—C84'—H84C	110.3
C40—C39—C38	118.7 (3)	C83'—C84'—H84C	110.3
C40—C39—C44	121.1 (3)	C85'—C84'—H84D	110.3
C38—C39—C44	120.3 (3)	C83'—C84'—H84D	110.3
C41—C40—C39	121.4 (4)	H84C—C84'—H84D	108.6
C41—C40—H40	119.3	O1'—C85'—C84'	102.7 (14)
C39—C40—H40	119.3	O1'—C85'—H85C	111.2
C40—C41—C42	120.0 (4)	C84'—C85'—H85C	111.2
C40—C41—H41	120.0	O1'—C85'—H85D	111.2
C42—C41—H41	120.0	C84'—C85'—H85D	111.2
C41—C42—C43	121.0 (4)	H85C—C85'—H85D	109.1
C41—C42—H42	119.5	C86—O2—C89	102.5 (11)
C43—C42—H42	119.5	O2—C86—C87	101.8 (13)
C42—C43—C38	118.6 (4)	O2—C86—H86A	111.4
C42—C43—C45	121.8 (4)	C87—C86—H86A	111.4
C38—C43—C45	119.6 (3)	O2—C86—H86B	111.4
C39—C44—H44A	109.5	C87—C86—H86B	111.4
C39—C44—H44B	109.5	H86A—C86—H86B	109.3
H44A—C44—H44B	109.5	C86—C87—C88	92.3 (13)
C39—C44—H44C	109.5	C86—C87—H87A	113.2
H44A—C44—H44C	109.5	C88—C87—H87A	113.2
H44B—C44—H44C	109.5	C86—C87—H87B	113.2
C43—C45—H45A	109.5	C88—C87—H87B	113.2
C43—C45—H45B	109.5	H87A—C87—H87B	110.6
H45A—C45—H45B	109.5	C87—C88—C89	95.5 (13)
C43—C45—H45C	109.5	C87—C88—H88A	112.6
H45A—C45—H45C	109.5	C89—C88—H88A	112.6
H45B—C45—H45C	109.5	C87—C88—H88B	112.6
N6—C46—C55	122.9 (2)	C89—C88—H88B	112.6
N6—C46—Fe1	118.09 (17)	H88A—C88—H88B	110.1
C55—C46—Fe1	118.73 (17)	O2—C89—C88	104.5 (11)
C46—N6—C37	116.62 (19)	O2—C89—H89A	110.9
C46—N6—C47	125.8 (2)	C88—C89—H89A	110.9
C37—N6—C47	116.94 (19)	O2—C89—H89B	110.9
C48—C47—C52	122.2 (3)	C88—C89—H89B	110.9
C48—C47—N6	118.0 (2)	H89A—C89—H89B	108.9
C52—C47—N6	119.8 (2)	C89'—O2'—C86'	99.2 (16)
C49—C48—C47	118.0 (3)	O2'—C86'—C87'	104.8 (16)
C49—C48—C53	119.9 (3)	O2'—C86'—H86C	110.8
C47—C48—C53	122.1 (3)	C87'—C86'—H86C	110.8
C50—C49—C48	120.7 (4)	O2'—C86'—H86D	110.8
C50—C49—H49	119.7	C87'—C86'—H86D	110.8
C48—C49—H49	119.7	H86C—C86'—H86D	108.9
C51—C50—C49	121.0 (4)	C88'—C87'—C86'	89.5 (15)
C51—C50—H50	119.5	C88'—C87'—H87C	113.7
C49—C50—H50	119.5	C86'—C87'—H87C	113.7
C50—C51—C52	120.9 (4)	C88'—C87'—H87D	113.7
C50—C51—H51	119.6	C86'—C87'—H87D	113.7
C52—C51—H51	119.6	H87C—C87'—H87D	111.0
C51—C52—C47	117.2 (3)	C87'—C88'—C89'	89.8 (15)
C51—C52—C54	121.5 (3)	C87'—C88'—H88C	113.7
C47—C52—C54	121.3 (3)	C89'—C88'—H88C	113.7
C48—C53—H53A	109.5	C87'—C88'—H88D	113.7
C48—C53—H53B	109.5	C89'—C88'—H88D	113.7
H53A—C53—H53B	109.5	H88C—C88'—H88D	110.9
C48—C53—H53C	109.5	O2'—C89'—C88'	99.6 (15)
H53A—C53—H53C	109.5	O2'—C89'—H89C	111.8
H53B—C53—H53C	109.5	C88'—C89'—H89C	111.8
C52—C54—H54A	109.5	O2'—C89'—H89D	111.8
C52—C54—H54B	109.5	C88'—C89'—H89D	111.8
H54A—C54—H54B	109.5	H89C—C89'—H89D	109.6
			
C10—Fe1—C1—N1	−34 (7)	C53—C48—C49—C50	178.5 (4)
C46—Fe1—C1—N1	146 (7)	C48—C49—C50—C51	0.0 (6)
C73—Fe1—C1—N1	64 (7)	C49—C50—C51—C52	0.8 (6)
C28—Fe1—C1—N1	−133 (7)	C50—C51—C52—C47	−0.6 (5)
C7—C2—C3—C4	−1 (3)	C50—C51—C52—C54	177.8 (3)
N1—C2—C3—C4	175.9 (18)	C48—C47—C52—C51	−0.5 (4)
C7—C2—C3—C8	178 (2)	N6—C47—C52—C51	179.0 (2)
N1—C2—C3—C8	−5 (3)	C48—C47—C52—C54	−178.9 (3)
C2—C3—C4—C5	0 (2)	N6—C47—C52—C54	0.6 (4)
C8—C3—C4—C5	−179 (2)	N6—C46—C55—C64	−168.7 (2)
C3—C4—C5—C6	2 (2)	Fe1—C46—C55—C64	5.4 (3)
C4—C5—C6—C7	−2 (2)	N6—C46—C55—N7	12.7 (4)
C5—C6—C7—C2	1 (2)	Fe1—C46—C55—N7	−173.24 (18)
C5—C6—C7—C9	175.2 (18)	C64—C55—N7—C56	−46.0 (4)
C3—C2—C7—C6	1 (3)	C46—C55—N7—C56	132.5 (3)
N1—C2—C7—C6	−176.5 (18)	C55—N7—C56—C61	−28.1 (4)
C3—C2—C7—C9	−172 (2)	C55—N7—C56—C57	154.8 (2)
N1—C2—C7—C9	10 (3)	N7—C56—C57—C58	−177.0 (3)
C7'—C2'—C3'—C4'	2 (2)	C61—C56—C57—C58	5.8 (4)
N1'—C2'—C3'—C4'	−175.7 (17)	N7—C56—C57—C62	4.0 (4)
C7'—C2'—C3'—C8'	−174 (2)	C61—C56—C57—C62	−173.2 (3)
N1'—C2'—C3'—C8'	8 (3)	C56—C57—C58—C59	−0.6 (5)
C2'—C3'—C4'—C5'	0 (2)	C62—C57—C58—C59	178.5 (3)
C8'—C3'—C4'—C5'	176 (2)	C57—C58—C59—C60	−3.7 (6)
C3'—C4'—C5'—C6'	−2.3 (19)	C58—C59—C60—C61	2.8 (6)
C4'—C5'—C6'—C7'	2.9 (19)	C59—C60—C61—C56	2.4 (5)
C5'—C6'—C7'—C2'	−1 (2)	C59—C60—C61—C63	−174.8 (3)
C5'—C6'—C7'—C9'	−174.8 (17)	N7—C56—C61—C60	176.3 (3)
C3'—C2'—C7'—C6'	−2 (2)	C57—C56—C61—C60	−6.7 (4)
N1'—C2'—C7'—C6'	176.2 (17)	N7—C56—C61—C63	−6.7 (4)
C3'—C2'—C7'—C9'	172.5 (19)	C57—C56—C61—C63	170.3 (3)
N1'—C2'—C7'—C9'	−9 (3)	C46—C55—C64—N8'	170.7 (13)
C16—C11—C12—C13	−0.3 (5)	N7—C55—C64—N8'	−10.6 (13)
N2—C11—C12—C13	178.9 (3)	C46—C55—C64—N8	164.7 (12)
C16—C11—C12—C17	179.4 (3)	N7—C55—C64—N8	−16.6 (12)
N2—C11—C12—C17	−1.4 (4)	C46—C55—C64—C73	−17.8 (3)
C11—C12—C13—C14	−0.5 (6)	N7—C55—C64—C73	160.9 (2)
C17—C12—C13—C14	179.8 (4)	C55—C64—N8—C65	0 (3)
C12—C13—C14—C15	0.6 (7)	C73—C64—N8—C65	−177.6 (17)
C13—C14—C15—C16	0.0 (6)	C64—N8—C65—C66	−64 (4)
N2—C11—C16—C15	−178.2 (3)	C64—N8—C65—C70	126 (3)
C12—C11—C16—C15	0.9 (5)	C70—C65—C66—C67	−4 (4)
N2—C11—C16—C18	1.2 (4)	N8—C65—C66—C67	−173 (3)
C12—C11—C16—C18	−179.6 (3)	C70—C65—C66—C71	170 (3)
C14—C15—C16—C11	−0.7 (5)	N8—C65—C66—C71	1 (4)
C14—C15—C16—C18	179.8 (3)	C65—C66—C67—C68	−1 (3)
C25—C20—C21—C22	0.0 (6)	C71—C66—C67—C68	−175.4 (17)
N3—C20—C21—C22	177.8 (4)	C66—C67—C68—C69	3 (2)
C25—C20—C21—C26	−177.7 (4)	C67—C68—C69—C70	1 (2)
N3—C20—C21—C26	0.1 (6)	C68—C69—C70—C65	−6 (3)
C20—C21—C22—C23	0.4 (8)	C68—C69—C70—C72	178.2 (19)
C26—C21—C22—C23	178.1 (6)	C66—C65—C70—C69	7 (4)
C21—C22—C23—C24	−0.1 (11)	N8—C65—C70—C69	177 (2)
C22—C23—C24—C25	−0.7 (10)	C66—C65—C70—C72	−177 (3)
C21—C20—C25—C24	−0.8 (6)	N8—C65—C70—C72	−7 (4)
N3—C20—C25—C24	−178.6 (3)	C55—C64—N8'—C65'	−26 (4)
C21—C20—C25—C27	178.6 (4)	C73—C64—N8'—C65'	162 (3)
N3—C20—C25—C27	0.8 (6)	C64—N8'—C65'—C66'	−40 (7)
C23—C24—C25—C20	1.1 (8)	C64—N8'—C65'—C70'	139 (3)
C23—C24—C25—C27	−178.2 (6)	C70'—C65'—C66'—C67'	−4 (6)
C37—C28—N4—C29	179.6 (2)	N8'—C65'—C66'—C67'	174 (4)
Fe1—C28—N4—C29	−7.5 (4)	C70'—C65'—C66'—C71'	179 (4)
C28—N4—C29—C34	−81.9 (3)	N8'—C65'—C66'—C71'	−2 (6)
C28—N4—C29—C30	107.2 (3)	C65'—C66'—C67'—C68'	6 (4)
C34—C29—C30—C31	4.1 (4)	C71'—C66'—C67'—C68'	−177 (2)
N4—C29—C30—C31	174.9 (3)	C66'—C67'—C68'—C69'	−4 (4)
C34—C29—C30—C35	−171.7 (3)	C67'—C68'—C69'—C70'	−2 (3)
N4—C29—C30—C35	−0.8 (4)	C68'—C69'—C70'—C65'	4 (4)
C29—C30—C31—C32	−1.3 (5)	C68'—C69'—C70'—C72'	177 (3)
C35—C30—C31—C32	174.4 (3)	C66'—C65'—C70'—C69'	−1 (6)
C30—C31—C32—C33	−1.9 (6)	N8'—C65'—C70'—C69'	−180 (3)
C31—C32—C33—C34	2.4 (5)	C66'—C65'—C70'—C72'	−174 (4)
C30—C29—C34—C33	−3.5 (4)	N8'—C65'—C70'—C72'	7 (6)
N4—C29—C34—C33	−174.3 (2)	N8'—C64—C73—N9	16.8 (13)
C30—C29—C34—C36	174.3 (3)	N8—C64—C73—N9	22.9 (9)
N4—C29—C34—C36	3.6 (4)	C55—C64—C73—N9	−155.2 (2)
C32—C33—C34—C29	0.3 (5)	N8'—C64—C73—Fe1	−166.2 (13)
C32—C33—C34—C36	−177.6 (3)	N8—C64—C73—Fe1	−160.1 (9)
N4—C28—C37—N5	−11.3 (4)	C55—C64—C73—Fe1	21.8 (2)
Fe1—C28—C37—N5	173.9 (2)	C64—C73—N9—C74	175.9 (2)
N4—C28—C37—N6	170.2 (2)	Fe1—C73—N9—C74	0.3 (4)
Fe1—C28—C37—N6	−4.6 (2)	C73—N9—C74—C75	−104.7 (3)
N6—C37—N5—C38	−179.2 (2)	C73—N9—C74—C79	86.6 (3)
C28—C37—N5—C38	2.5 (5)	C79—C74—C75—C76	−2.3 (4)
C37—N5—C38—C39	−95.2 (3)	N9—C74—C75—C76	−170.9 (2)
C37—N5—C38—C43	96.0 (3)	C79—C74—C75—C80	174.5 (3)
C43—C38—C39—C40	−2.6 (4)	N9—C74—C75—C80	5.8 (4)
N5—C38—C39—C40	−171.2 (3)	C74—C75—C76—C77	0.6 (5)
C43—C38—C39—C44	176.0 (3)	C80—C75—C76—C77	−176.2 (3)
N5—C38—C39—C44	7.4 (4)	C75—C76—C77—C78	0.2 (5)
C38—C39—C40—C41	1.3 (6)	C76—C77—C78—C79	0.7 (5)
C44—C39—C40—C41	−177.3 (4)	C77—C78—C79—C74	−2.3 (4)
C39—C40—C41—C42	0.8 (7)	C77—C78—C79—C81	173.0 (3)
C40—C41—C42—C43	−1.6 (7)	C75—C74—C79—C78	3.1 (4)
C41—C42—C43—C38	0.2 (6)	N9—C74—C79—C78	171.4 (2)
C41—C42—C43—C45	179.4 (4)	C75—C74—C79—C81	−172.1 (2)
C39—C38—C43—C42	1.9 (5)	N9—C74—C79—C81	−3.8 (4)
N5—C38—C43—C42	170.6 (3)	C85—O1—C82—C83	18 (3)
C39—C38—C43—C45	−177.3 (3)	O1—C82—C83—C84	0 (2)
N5—C38—C43—C45	−8.6 (4)	C82—C83—C84—C85	−17 (2)
C55—C46—N6—C37	178.3 (2)	C82—O1—C85—C84	−29 (3)
Fe1—C46—N6—C37	4.1 (3)	C83—C84—C85—O1	27 (2)
C55—C46—N6—C47	7.4 (4)	C85'—O1'—C82'—C83'	−36 (3)
Fe1—C46—N6—C47	−166.72 (18)	O1'—C82'—C83'—C84'	37 (3)
N5—C37—N6—C46	−178.2 (2)	C82'—C83'—C84'—C85'	−27 (3)
C28—C37—N6—C46	0.5 (3)	C82'—O1'—C85'—C84'	19 (4)
N5—C37—N6—C47	−6.5 (3)	C83'—C84'—C85'—O1'	5 (4)
C28—C37—N6—C47	172.2 (2)	C89—O2—C86—C87	33 (2)
C46—N6—C47—C48	83.3 (3)	O2—C86—C87—C88	−60 (2)
C37—N6—C47—C48	−87.5 (3)	C86—C87—C88—C89	60 (2)
C46—N6—C47—C52	−96.2 (3)	C86—O2—C89—C88	6 (2)
C37—N6—C47—C52	93.0 (3)	C87—C88—C89—O2	−43 (2)
C52—C47—C48—C49	1.2 (4)	C89'—O2'—C86'—C87'	−11 (3)
N6—C47—C48—C49	−178.3 (3)	O2'—C86'—C87'—C88'	51 (3)
C52—C47—C48—C53	−178.2 (3)	C86'—C87'—C88'—C89'	−67 (2)
N6—C47—C48—C53	2.3 (4)	C86'—O2'—C89'—C88'	−33 (3)
C47—C48—C49—C50	−1.0 (5)	C87'—C88'—C89'—O2'	67 (2)

### {5,6-Bis(2,6-dimethylanilino)-3-(2,6-dimethylphenyl)-1,2,7-tris[(2,6-dimethylphenyl)imino]-3-azoniahept-3-ene-1,4,7-triido}tris(2,6-dimethylphenyl isocyanide)iron tetrahydrofuran disolvate (2) Hydrogen-bond geometry (Å, º)

**Table:**

*D*—H···*A*	*D*—H	H···*A*	*D*···*A*	*D*—H···*A*
N8—H8···N9	0.86	2.10	2.515 (13)	109
N8′—H8′···N9	0.86	2.22	2.644 (18)	110

### (7-Methylindolin-1-ido-κN)(1,4,7,10,13,16-hexaoxacyclooctadecane-κ6O)potassium (3) Crystal data

**Table:**

[K(C_9_H_8_N)(C_12_H_24_O_6_)]	*F*(000) = 928
*M**_r_* = 433.57	*D*_x_ = 1.258 Mg m^−^^3^
Monoclinic, *P*2_1_/*n*	Mo *K*α radiation, λ = 0.71073 Å
*a* = 10.784 (3) Å	Cell parameters from 937 reflections
*b* = 9.754 (3) Å	θ = 2.8–25.0°
*c* = 21.783 (7) Å	µ = 0.27 mm^−^^1^
β = 91.864 (4)°	*T* = 173 K
*V* = 2290.1 (12) Å^3^	Block, red-brown
*Z* = 4	0.24 × 0.18 × 0.15 mm

### (7-Methylindolin-1-ido-κN)(1,4,7,10,13,16-hexaoxacyclooctadecane-κ6O)potassium (3) Data collection

**Table:**

Bruker SMART CCD platform diffractometer	2902 reflections with *I* > 2σ(*I*)
Radiation source: fine-focus sealed tube	*R*_int_ = 0.062
ω scans	θ_max_ = 25.1°, θ_min_ = 1.9°
Absorption correction: multi-scan (SADABS; Krause *et al.*, 2015)	*h* = −12→12
*T*_min_ = 0.843, *T*_max_ = 1.000	*k* = −11→11
21112 measured reflections	*l* = −25→25
4071 independent reflections	

### (7-Methylindolin-1-ido-κN)(1,4,7,10,13,16-hexaoxacyclooctadecane-κ6O)potassium (3) Refinement

**Table:**

Refinement on *F*^2^	Primary atom site location: structure-invariant direct methods
Least-squares matrix: full	Secondary atom site location: difference Fourier map
*R*[*F*^2^ > 2σ(*F*^2^)] = 0.059	Hydrogen site location: inferred from neighbouring sites
*wR*(*F*^2^) = 0.170	H-atom parameters constrained
*S* = 1.05	*w* = 1/[σ^2^(*F*_o_^2^) + (0.0838*P*)^2^ + 1.6493*P*] where *P* = (*F*_o_^2^ + 2*F*_c_^2^)/3
4071 reflections	(Δ/σ)_max_ < 0.001
355 parameters	Δρ_max_ = 0.58 e Å^−^^3^
195 restraints	Δρ_min_ = −0.23 e Å^−^^3^

### (7-Methylindolin-1-ido-κN)(1,4,7,10,13,16-hexaoxacyclooctadecane-κ6O)potassium (3) Special details

**Table:**

Geometry. All esds (except the esd in the dihedral angle between two l.s. planes) are estimated using the full covariance matrix. The cell esds are taken into account individually in the estimation of esds in distances, angles and torsion angles; correlations between esds in cell parameters are only used when they are defined by crystal symmetry. An approximate (isotropic) treatment of cell esds is used for estimating esds involving l.s. planes.
Refinement. The 18-crown-6 macrocycle is also disordered (see below). The eight largest residual peaks are the two peaks near the K atom and those for six O atoms of the minor component of disorder. However, the data-to-parameter ratio drops below eight if this disorder is modeled. Thus only the anion disorder was modeled.The anion is modeled as disordered with the planar flip of itself (0.905 (3):0.095 (3)). Analogous bond lengths and angles between the two positions of the disordered anion were restrained to be similar. Anisotropic displacement parameters for proximal atoms were restrained to be similar and restrained toward the expected motion relative to bond direction.

### (7-Methylindolin-1-ido-κN)(1,4,7,10,13,16-hexaoxacyclooctadecane-κ6O)potassium (3) Fractional atomic coordinates and isotropic or equivalent isotropic displacement parameters (Å^2^)

**Table:**

	*x*	*y*	*z*	*U*_iso_*/*U*_eq_	Occ. (<1)
K1	0.47867 (6)	0.10916 (7)	0.68763 (3)	0.0402 (2)	
N1	0.6799 (3)	0.1406 (3)	0.61289 (13)	0.0430 (7)	0.905 (3)
C1	0.7705 (4)	0.0421 (5)	0.61419 (19)	0.0459 (10)	0.905 (3)
H1	0.765129	−0.039544	0.637708	0.055*	0.905 (3)
C2	0.7249 (8)	0.2379 (9)	0.5741 (6)	0.0359 (8)	0.905 (3)
C3	0.6675 (3)	0.3618 (4)	0.55605 (16)	0.0432 (9)	0.905 (3)
C4	0.7309 (4)	0.4449 (4)	0.51661 (17)	0.0515 (9)	0.905 (3)
H4	0.694709	0.529528	0.504037	0.062*	0.905 (3)
C5	0.8463 (4)	0.4090 (4)	0.49457 (19)	0.0576 (10)	0.905 (3)
H5	0.886958	0.469650	0.467653	0.069*	0.905 (3)
C6	0.9020 (4)	0.2882 (5)	0.5110 (2)	0.0539 (11)	0.905 (3)
H6	0.980133	0.264237	0.495166	0.065*	0.905 (3)
C7	0.8427 (3)	0.1996 (4)	0.55146 (15)	0.0427 (8)	0.905 (3)
C8	0.5449 (6)	0.3985 (6)	0.5809 (4)	0.0589 (13)	0.905 (3)
H8A	0.517177	0.486450	0.563550	0.088*	0.905 (3)
H8B	0.552535	0.406093	0.625736	0.088*	0.905 (3)
H8C	0.484182	0.327207	0.569810	0.088*	0.905 (3)
C9	0.8698 (3)	0.0718 (4)	0.57852 (17)	0.0470 (9)	0.905 (3)
H9	0.941701	0.017349	0.573247	0.056*	0.905 (3)
N1'	0.6079 (19)	0.246 (2)	0.6119 (10)	0.034 (5)	0.095 (3)
C1'	0.567 (5)	0.370 (5)	0.588 (3)	0.053 (7)	0.095 (3)
H1'	0.491531	0.409632	0.599970	0.064*	0.095 (3)
C2'	0.716 (8)	0.228 (9)	0.581 (6)	0.038 (3)	0.095 (3)
C3'	0.800 (2)	0.119 (3)	0.5895 (13)	0.042 (3)	0.095 (3)
C4'	0.904 (3)	0.121 (3)	0.5543 (14)	0.045 (3)	0.095 (3)
H4'	0.961602	0.047874	0.557570	0.053*	0.095 (3)
C5'	0.927 (3)	0.229 (4)	0.514 (2)	0.056 (5)	0.095 (3)
H5'	0.999937	0.226918	0.490635	0.067*	0.095 (3)
C6'	0.849 (3)	0.337 (4)	0.5074 (19)	0.054 (4)	0.095 (3)
H6'	0.865659	0.409039	0.479391	0.065*	0.095 (3)
C7'	0.741 (2)	0.342 (3)	0.5425 (14)	0.046 (3)	0.095 (3)
C8'	0.777 (4)	0.011 (4)	0.6371 (18)	0.045 (7)	0.095 (3)
H8D	0.849934	−0.047214	0.642319	0.068*	0.095 (3)
H8E	0.705141	−0.044390	0.623772	0.068*	0.095 (3)
H8F	0.759299	0.056058	0.676182	0.068*	0.095 (3)
C9'	0.642 (3)	0.432 (3)	0.5462 (15)	0.047 (3)	0.095 (3)
H9'	0.628112	0.516371	0.525073	0.056*	0.095 (3)
O1	0.4936 (3)	0.2527 (4)	0.80078 (13)	0.0813 (9)	
C10	0.5423 (5)	0.1834 (6)	0.85294 (19)	0.0962 (18)	
H10A	0.473722	0.140953	0.875365	0.115*	
H10B	0.584995	0.249668	0.880795	0.115*	
C11	0.6316 (5)	0.0750 (7)	0.8346 (2)	0.0989 (18)	
H11A	0.700394	0.117054	0.812171	0.119*	
H11B	0.667180	0.028987	0.871642	0.119*	
O2	0.5698 (3)	−0.0206 (3)	0.79708 (12)	0.0761 (8)	
C12	0.6427 (5)	−0.1387 (6)	0.7868 (2)	0.1005 (19)	
H12A	0.663983	−0.184421	0.826332	0.121*	
H12B	0.720798	−0.112539	0.767181	0.121*	
C13	0.5695 (5)	−0.2339 (5)	0.7458 (2)	0.0908 (16)	
H13A	0.616207	−0.320085	0.740140	0.109*	
H13B	0.489695	−0.256513	0.764607	0.109*	
O3	0.5482 (3)	−0.1709 (3)	0.69021 (12)	0.0670 (7)	
C14	0.4844 (5)	−0.2566 (4)	0.6477 (3)	0.0919 (15)	
H14A	0.404047	−0.284974	0.664362	0.110*	
H14B	0.534049	−0.340079	0.640399	0.110*	
C15	0.4630 (5)	−0.1815 (5)	0.5893 (2)	0.0866 (14)	
H15A	0.541755	−0.141718	0.575414	0.104*	
H15B	0.430216	−0.244508	0.557041	0.104*	
O4	0.3765 (2)	−0.0765 (3)	0.59986 (12)	0.0652 (7)	
C16	0.3562 (4)	0.0063 (4)	0.54769 (16)	0.0627 (10)	
H16A	0.336048	−0.051851	0.511466	0.075*	
H16B	0.432230	0.059113	0.539340	0.075*	
C17	0.2516 (4)	0.1019 (5)	0.55876 (17)	0.0686 (11)	
H17A	0.232187	0.155687	0.521127	0.082*	
H17B	0.176785	0.049111	0.569190	0.082*	
O5	0.2850 (2)	0.1903 (3)	0.60716 (11)	0.0604 (7)	
C18	0.1871 (3)	0.2743 (4)	0.62523 (19)	0.0658 (10)	
H18A	0.123904	0.218585	0.645761	0.079*	
H18B	0.147260	0.318732	0.588798	0.079*	
C19	0.2377 (4)	0.3800 (4)	0.6681 (2)	0.0797 (13)	
H19A	0.300098	0.436117	0.647146	0.096*	
H19B	0.170037	0.441474	0.680668	0.096*	
O6	0.2930 (3)	0.3180 (3)	0.72029 (15)	0.0772 (8)	
C20	0.3488 (5)	0.4134 (5)	0.7604 (3)	0.112 (2)	
H20A	0.286899	0.482739	0.772129	0.134*	
H20B	0.416627	0.461167	0.739537	0.134*	
C21	0.3987 (6)	0.3447 (8)	0.8154 (3)	0.131 (3)	
H21A	0.431752	0.414059	0.844821	0.157*	
H21B	0.331291	0.294250	0.835385	0.157*	

### (7-Methylindolin-1-ido-κN)(1,4,7,10,13,16-hexaoxacyclooctadecane-κ6O)potassium (3) Atomic displacement parameters (Å^2^)

**Table:**

	*U*^11^	*U*^22^	*U*^33^	*U*^12^	*U*^13^	*U*^23^
K1	0.0465 (4)	0.0362 (4)	0.0378 (4)	−0.0007 (3)	0.0011 (3)	−0.0012 (3)
N1	0.0440 (16)	0.0398 (16)	0.0456 (16)	−0.0050 (13)	0.0062 (13)	−0.0020 (12)
C1	0.051 (2)	0.043 (2)	0.044 (3)	−0.0024 (19)	−0.0018 (19)	0.0023 (18)
C2	0.037 (2)	0.035 (2)	0.036 (4)	−0.006 (2)	−0.0028 (14)	−0.0074 (15)
C3	0.047 (2)	0.038 (2)	0.0442 (19)	−0.0057 (17)	−0.0059 (16)	−0.0084 (16)
C4	0.062 (2)	0.0396 (19)	0.052 (2)	−0.0041 (17)	−0.0126 (18)	0.0030 (16)
C5	0.059 (2)	0.060 (3)	0.053 (2)	−0.015 (2)	−0.0036 (18)	0.015 (2)
C6	0.045 (3)	0.069 (3)	0.048 (2)	−0.009 (2)	0.003 (2)	0.010 (2)
C7	0.0396 (18)	0.049 (2)	0.0391 (18)	−0.0058 (16)	−0.0033 (14)	0.0011 (15)
C8	0.062 (3)	0.041 (3)	0.074 (4)	0.010 (2)	0.004 (3)	−0.005 (2)
C9	0.040 (2)	0.052 (2)	0.049 (2)	0.0037 (16)	−0.0017 (16)	0.0041 (17)
N1'	0.037 (10)	0.025 (9)	0.039 (12)	−0.006 (7)	−0.002 (8)	−0.008 (7)
C1'	0.059 (13)	0.032 (13)	0.069 (15)	0.003 (9)	0.004 (12)	0.005 (11)
C2'	0.038 (6)	0.036 (6)	0.040 (7)	−0.004 (5)	0.000 (6)	−0.005 (6)
C3'	0.041 (6)	0.043 (6)	0.042 (7)	0.001 (5)	−0.004 (6)	−0.002 (6)
C4'	0.040 (6)	0.050 (7)	0.043 (7)	−0.002 (6)	−0.004 (6)	0.001 (6)
C5'	0.051 (9)	0.067 (9)	0.050 (9)	−0.004 (7)	−0.002 (8)	0.012 (8)
C6'	0.051 (6)	0.061 (7)	0.049 (7)	−0.007 (6)	0.000 (6)	0.016 (6)
C7'	0.051 (6)	0.042 (6)	0.044 (7)	−0.007 (5)	−0.005 (5)	−0.001 (6)
C8'	0.039 (14)	0.048 (13)	0.049 (16)	0.021 (11)	0.012 (12)	0.004 (10)
C9'	0.053 (6)	0.038 (6)	0.048 (7)	−0.005 (6)	−0.008 (6)	−0.001 (6)
O1	0.0748 (19)	0.103 (2)	0.0658 (18)	−0.0092 (17)	0.0074 (14)	−0.0332 (16)
C10	0.104 (4)	0.142 (5)	0.041 (2)	−0.052 (4)	−0.001 (2)	−0.022 (3)
C11	0.083 (3)	0.158 (5)	0.055 (3)	−0.017 (4)	−0.019 (2)	0.014 (3)
O2	0.0715 (18)	0.104 (2)	0.0522 (15)	0.0060 (17)	−0.0033 (13)	0.0143 (15)
C12	0.099 (4)	0.136 (5)	0.067 (3)	0.059 (4)	−0.001 (3)	0.038 (3)
C13	0.117 (4)	0.070 (3)	0.087 (3)	0.039 (3)	0.030 (3)	0.035 (3)
O3	0.0851 (19)	0.0446 (14)	0.0720 (17)	0.0067 (13)	0.0135 (14)	0.0112 (13)
C14	0.110 (4)	0.035 (2)	0.130 (4)	0.002 (2)	−0.002 (3)	−0.013 (3)
C15	0.111 (4)	0.056 (3)	0.092 (3)	0.006 (3)	−0.002 (3)	−0.032 (2)
O4	0.0704 (17)	0.0560 (15)	0.0691 (17)	−0.0098 (13)	0.0028 (13)	−0.0144 (13)
C16	0.064 (2)	0.080 (3)	0.0434 (19)	−0.008 (2)	−0.0008 (16)	−0.0116 (19)
C17	0.056 (2)	0.099 (3)	0.050 (2)	−0.016 (2)	−0.0069 (17)	0.002 (2)
O5	0.0475 (14)	0.0681 (16)	0.0655 (16)	0.0041 (12)	−0.0002 (11)	0.0055 (13)
C18	0.048 (2)	0.065 (2)	0.085 (3)	0.0133 (18)	0.0041 (19)	0.017 (2)
C19	0.052 (2)	0.059 (2)	0.128 (4)	0.010 (2)	0.008 (2)	0.007 (3)
O6	0.0682 (18)	0.0612 (17)	0.102 (2)	0.0043 (14)	0.0066 (16)	−0.0249 (16)
C20	0.084 (3)	0.083 (4)	0.167 (6)	0.017 (3)	−0.018 (4)	−0.078 (4)
C21	0.090 (4)	0.162 (6)	0.141 (5)	0.013 (4)	0.001 (4)	−0.104 (5)

### (7-Methylindolin-1-ido-κN)(1,4,7,10,13,16-hexaoxacyclooctadecane-κ6O)potassium (3) Geometric parameters (Å, º)

**Table:**

K1—N1'	2.571 (19)	C8'—H8E	0.9800
K1—N1	2.772 (3)	C8'—H8F	0.9800
K1—O5	2.797 (2)	C9'—H9'	0.9500
K1—O4	2.831 (3)	O1—C21	1.406 (6)
K1—O3	2.832 (3)	O1—C10	1.409 (6)
K1—O1	2.835 (3)	C10—C11	1.493 (8)
K1—O2	2.846 (3)	C10—H10A	0.9900
K1—O6	2.959 (3)	C10—H10B	0.9900
K1—C16	3.432 (4)	C11—O2	1.395 (6)
K1—C1'	3.49 (3)	C11—H11A	0.9900
N1—C1	1.369 (5)	C11—H11B	0.9900
N1—C2	1.370 (5)	O2—C12	1.418 (6)
C1—C9	1.374 (6)	C12—C13	1.495 (8)
C1—H1	0.9500	C12—H12A	0.9900
C2—C3	1.408 (6)	C12—H12B	0.9900
C2—C7	1.427 (5)	C13—O3	1.372 (5)
C3—C4	1.379 (5)	C13—H13A	0.9900
C3—C8	1.489 (6)	C13—H13B	0.9900
C4—C5	1.393 (6)	O3—C14	1.410 (5)
C4—H4	0.9500	C14—C15	1.480 (7)
C5—C6	1.365 (6)	C14—H14A	0.9900
C5—H5	0.9500	C14—H14B	0.9900
C6—C7	1.403 (5)	C15—O4	1.408 (5)
C6—H6	0.9500	C15—H15A	0.9900
C7—C9	1.405 (5)	C15—H15B	0.9900
C8—H8A	0.9800	O4—C16	1.406 (5)
C8—H8B	0.9800	C16—C17	1.489 (6)
C8—H8C	0.9800	C16—H16A	0.9900
C9—H9	0.9500	C16—H16B	0.9900
N1'—C1'	1.374 (19)	C17—O5	1.400 (4)
N1'—C2'	1.374 (18)	C17—H17A	0.9900
C1'—C9'	1.375 (11)	C17—H17B	0.9900
C1'—H1'	0.9500	O5—C18	1.403 (4)
C2'—C3'	1.407 (19)	C18—C19	1.484 (6)
C2'—C7'	1.429 (18)	C18—H18A	0.9900
C3'—C4'	1.376 (18)	C18—H18B	0.9900
C3'—C8'	1.500 (18)	C19—O6	1.403 (5)
C4'—C5'	1.398 (19)	C19—H19A	0.9900
C4'—H4'	0.9500	C19—H19B	0.9900
C5'—C6'	1.358 (19)	O6—C20	1.399 (5)
C5'—H5'	0.9500	C20—C21	1.460 (9)
C6'—C7'	1.406 (18)	C20—H20A	0.9900
C6'—H6'	0.9500	C20—H20B	0.9900
C7'—C9'	1.396 (18)	C21—H21A	0.9900
C8'—H8D	0.9800	C21—H21B	0.9900
			
N1'—K1—O5	81.9 (5)	C3'—C8'—H8D	109.5
N1—K1—O5	100.57 (8)	C3'—C8'—H8E	109.5
N1'—K1—O4	96.1 (5)	H8D—C8'—H8E	109.5
N1—K1—O4	88.22 (8)	C3'—C8'—H8F	109.5
O5—K1—O4	59.44 (8)	H8D—C8'—H8F	109.5
N1'—K1—O3	111.5 (4)	H8E—C8'—H8F	109.5
N1—K1—O3	84.63 (8)	C1'—C9'—C7'	103.0 (17)
O5—K1—O3	118.50 (8)	C1'—C9'—H9'	128.5
O4—K1—O3	59.55 (8)	C7'—C9'—H9'	128.5
N1'—K1—O1	106.5 (5)	C21—O1—C10	112.2 (5)
N1—K1—O1	115.63 (9)	C21—O1—K1	119.4 (3)
O5—K1—O1	115.17 (9)	C10—O1—K1	118.2 (3)
O4—K1—O1	155.96 (9)	O1—C10—C11	110.5 (4)
O3—K1—O1	116.85 (10)	O1—C10—H10A	109.5
N1'—K1—O2	126.2 (5)	C11—C10—H10A	109.5
N1—K1—O2	106.60 (9)	O1—C10—H10B	109.5
O5—K1—O2	151.81 (8)	C11—C10—H10B	109.5
O4—K1—O2	113.42 (9)	H10A—C10—H10B	108.1
O3—K1—O2	58.04 (9)	O2—C11—C10	109.2 (4)
O1—K1—O2	58.86 (10)	O2—C11—H11A	109.8
N1'—K1—O6	100.4 (4)	C10—C11—H11A	109.8
N1—K1—O6	127.88 (9)	O2—C11—H11B	109.8
O5—K1—O6	57.22 (8)	C10—C11—H11B	109.8
O4—K1—O6	110.57 (8)	H11A—C11—H11B	108.3
O3—K1—O6	147.10 (8)	C11—O2—C12	112.3 (4)
O1—K1—O6	58.04 (9)	C11—O2—K1	109.8 (3)
O2—K1—O6	108.81 (9)	C12—O2—K1	114.0 (2)
N1'—K1—C16	77.5 (5)	O2—C12—C13	108.3 (4)
N1—K1—C16	78.26 (9)	O2—C12—H12A	110.0
O5—K1—C16	42.97 (9)	C13—C12—H12A	110.0
O4—K1—C16	23.51 (9)	O2—C12—H12B	110.0
O3—K1—C16	80.18 (9)	C13—C12—H12B	110.0
O1—K1—C16	157.82 (10)	H12A—C12—H12B	108.4
O2—K1—C16	136.59 (10)	O3—C13—C12	108.6 (4)
O6—K1—C16	99.85 (9)	O3—C13—H13A	110.0
N1'—K1—C1'	19.5 (5)	C12—C13—H13A	110.0
O5—K1—C1'	67.6 (12)	O3—C13—H13B	110.0
O4—K1—C1'	98.9 (16)	C12—C13—H13B	110.0
O3—K1—C1'	129.5 (8)	H13A—C13—H13B	108.3
O1—K1—C1'	99.9 (15)	C13—O3—C14	112.3 (4)
O2—K1—C1'	138.5 (14)	C13—O3—K1	119.1 (3)
O6—K1—C1'	81.4 (5)	C14—O3—K1	115.9 (2)
C16—K1—C1'	76.5 (16)	O3—C14—C15	109.3 (4)
C1—N1—C2	103.4 (3)	O3—C14—H14A	109.8
C1—N1—K1	118.8 (2)	C15—C14—H14A	109.8
C2—N1—K1	137.6 (2)	O3—C14—H14B	109.8
N1—C1—C9	114.1 (4)	C15—C14—H14B	109.8
N1—C1—H1	122.9	H14A—C14—H14B	108.3
C9—C1—H1	122.9	O4—C15—C14	107.8 (4)
N1—C2—C3	127.3 (3)	O4—C15—H15A	110.2
N1—C2—C7	111.6 (3)	C14—C15—H15A	110.2
C3—C2—C7	121.1 (3)	O4—C15—H15B	110.2
C4—C3—C2	117.1 (4)	C14—C15—H15B	110.2
C4—C3—C8	123.3 (4)	H15A—C15—H15B	108.5
C2—C3—C8	119.5 (4)	C16—O4—C15	111.8 (3)
C3—C4—C5	122.3 (4)	C16—O4—K1	103.0 (2)
C3—C4—H4	118.9	C15—O4—K1	109.3 (2)
C5—C4—H4	118.9	O4—C16—C17	109.1 (3)
C6—C5—C4	121.1 (4)	O4—C16—K1	53.46 (16)
C6—C5—H5	119.4	C17—C16—K1	86.8 (2)
C4—C5—H5	119.4	O4—C16—H16A	109.9
C5—C6—C7	119.4 (4)	C17—C16—H16A	109.9
C5—C6—H6	120.3	K1—C16—H16A	160.6
C7—C6—H6	120.3	O4—C16—H16B	109.9
C6—C7—C9	135.8 (4)	C17—C16—H16B	109.9
C6—C7—C2	119.0 (4)	K1—C16—H16B	73.4
C9—C7—C2	105.2 (3)	H16A—C16—H16B	108.3
C3—C8—H8A	109.5	O5—C17—C16	109.3 (3)
C3—C8—H8B	109.5	O5—C17—H17A	109.8
H8A—C8—H8B	109.5	C16—C17—H17A	109.8
C3—C8—H8C	109.5	O5—C17—H17B	109.8
H8A—C8—H8C	109.5	C16—C17—H17B	109.8
H8B—C8—H8C	109.5	H17A—C17—H17B	108.3
C1—C9—C7	105.6 (4)	C17—O5—C18	113.2 (3)
C1—C9—H9	127.2	C17—O5—K1	117.8 (2)
C7—C9—H9	127.2	C18—O5—K1	122.9 (2)
C1'—N1'—C2'	101.3 (16)	O5—C18—C19	108.5 (3)
C1'—N1'—K1	121.7 (14)	O5—C18—H18A	110.0
C2'—N1'—K1	136.8 (15)	C19—C18—H18A	110.0
N1'—C1'—C9'	117.0 (19)	O5—C18—H18B	110.0
N1'—C1'—K1	38.8 (10)	C19—C18—H18B	110.0
C9'—C1'—K1	155.7 (16)	H18A—C18—H18B	108.4
N1'—C1'—H1'	121.5	O6—C19—C18	110.4 (3)
C9'—C1'—H1'	121.5	O6—C19—H19A	109.6
K1—C1'—H1'	82.8	C18—C19—H19A	109.6
N1'—C2'—C3'	126 (2)	O6—C19—H19B	109.6
N1'—C2'—C7'	111.5 (17)	C18—C19—H19B	109.6
C3'—C2'—C7'	121.9 (19)	H19A—C19—H19B	108.1
C4'—C3'—C2'	116.6 (18)	C20—O6—C19	112.4 (4)
C4'—C3'—C8'	124 (2)	C20—O6—K1	109.1 (3)
C2'—C3'—C8'	119 (2)	C19—O6—K1	112.0 (2)
C3'—C4'—C5'	122 (2)	O6—C20—C21	110.3 (5)
C3'—C4'—H4'	119.0	O6—C20—H20A	109.6
C5'—C4'—H4'	119.0	C21—C20—H20A	109.6
C6'—C5'—C4'	122 (2)	O6—C20—H20B	109.6
C6'—C5'—H5'	119.1	C21—C20—H20B	109.6
C4'—C5'—H5'	119.1	H20A—C20—H20B	108.1
C5'—C6'—C7'	119 (2)	O1—C21—C20	111.0 (5)
C5'—C6'—H6'	120.4	O1—C21—H21A	109.4
C7'—C6'—H6'	120.4	C20—C21—H21A	109.4
C9'—C7'—C6'	135 (2)	O1—C21—H21B	109.4
C9'—C7'—C2'	107.1 (16)	C20—C21—H21B	109.4
C6'—C7'—C2'	118.2 (18)	H21A—C21—H21B	108.0
			
C2—N1—C1—C9	0.0 (8)	N1'—C2'—C7'—C9'	−3 (14)
K1—N1—C1—C9	−176.0 (3)	C3'—C2'—C7'—C9'	−178 (11)
C1—N1—C2—C3	179.4 (12)	N1'—C2'—C7'—C6'	−179 (8)
K1—N1—C2—C3	−6 (2)	C3'—C2'—C7'—C6'	6 (18)
C1—N1—C2—C7	0.1 (12)	N1'—C1'—C9'—C7'	−1 (8)
K1—N1—C2—C7	174.9 (3)	K1—C1'—C9'—C7'	−4 (15)
N1—C2—C3—C4	179.6 (10)	C6'—C7'—C9'—C1'	177 (6)
C7—C2—C3—C4	−1.1 (16)	C2'—C7'—C9'—C1'	2 (9)
N1—C2—C3—C8	1.0 (18)	C21—O1—C10—C11	173.2 (5)
C7—C2—C3—C8	−179.7 (9)	K1—O1—C10—C11	27.9 (5)
C2—C3—C4—C5	0.7 (9)	O1—C10—C11—O2	−61.3 (5)
C8—C3—C4—C5	179.2 (5)	C10—C11—O2—C12	−169.5 (4)
C3—C4—C5—C6	0.4 (6)	C10—C11—O2—K1	62.5 (4)
C4—C5—C6—C7	−0.9 (6)	C11—O2—C12—C13	−179.4 (4)
C5—C6—C7—C9	−179.4 (4)	K1—O2—C12—C13	−53.7 (5)
C5—C6—C7—C2	0.5 (9)	O2—C12—C13—O3	63.4 (5)
N1—C2—C7—C6	180.0 (7)	C12—C13—O3—C14	177.2 (4)
C3—C2—C7—C6	0.6 (16)	C12—C13—O3—K1	−42.7 (5)
N1—C2—C7—C9	−0.2 (12)	C13—O3—C14—C15	179.1 (4)
C3—C2—C7—C9	−179.6 (10)	K1—O3—C14—C15	37.7 (5)
N1—C1—C9—C7	−0.1 (5)	O3—C14—C15—O4	−68.6 (5)
C6—C7—C9—C1	180.0 (4)	C14—C15—O4—C16	176.2 (3)
C2—C7—C9—C1	0.2 (8)	C14—C15—O4—K1	62.8 (4)
C2'—N1'—C1'—C9'	−1 (11)	C15—O4—C16—C17	172.0 (3)
K1—N1'—C1'—C9'	−178 (5)	K1—O4—C16—C17	−70.7 (3)
C2'—N1'—C1'—K1	176 (9)	C15—O4—C16—K1	−117.2 (3)
C1'—N1'—C2'—C3'	177 (14)	O4—C16—C17—O5	63.9 (4)
K1—N1'—C2'—C3'	−7 (23)	K1—C16—C17—O5	14.5 (3)
C1'—N1'—C2'—C7'	3 (13)	C16—C17—O5—C18	−173.5 (3)
K1—N1'—C2'—C7'	178 (3)	C16—C17—O5—K1	−20.2 (4)
N1'—C2'—C3'—C4'	−179 (11)	C17—O5—C18—C19	−169.7 (3)
C7'—C2'—C3'—C4'	−5 (17)	K1—O5—C18—C19	38.6 (4)
N1'—C2'—C3'—C8'	−2 (19)	O5—C18—C19—O6	−60.4 (4)
C7'—C2'—C3'—C8'	172 (10)	C18—C19—O6—C20	176.4 (4)
C2'—C3'—C4'—C5'	2 (10)	C18—C19—O6—K1	53.1 (4)
C8'—C3'—C4'—C5'	−175 (4)	C19—O6—C20—C21	176.8 (4)
C3'—C4'—C5'—C6'	0 (8)	K1—O6—C20—C21	−58.3 (5)
C4'—C5'—C6'—C7'	1 (9)	C10—O1—C21—C20	−179.1 (5)
C5'—C6'—C7'—C9'	−178 (5)	K1—O1—C21—C20	−34.3 (7)
C5'—C6'—C7'—C2'	−4 (11)	O6—C20—C21—O1	63.7 (7)
